# Mechanical rolling formation of interpenetrated lithium metal/lithium tin alloy foil for ultrahigh-rate battery anode

**DOI:** 10.1038/s41467-020-14550-3

**Published:** 2020-02-11

**Authors:** Mintao Wan, Sujin Kang, Li Wang, Hyun-Wook Lee, Guangyuan Wesley Zheng, Yi Cui, Yongming Sun

**Affiliations:** 1 Wuhan National Laboratory for Optoelectronics (WNLO), Huazhong University of Science and Technology (HUST), Wuhan, China; 2 School of Energy and Chemical Engineering, Ulsan National Institute of Science and Technology (UNIST), 50 UNIST-gil, Ulsan, 44919 Korea; 3 Institute of Nuclear & New Energy Technology, Tsinghua University, Beijing, 100084 China; 4 Institute of Materials Research and Engineering, A*STAR, 2 Fusionopolis Way, Innovis, Singapore, 138634 Singapore; 5 Department of Chemical and Biomolecular Engineering, National University of Singapore, 10 Kent Ridge Crescent, Singapore, 119260 Singapore; 6 Department of Materials Science and Engineering, Stanford University, Stanford, CA 94305 USA; 7 Stanford Institute for Materials and Energy Sciences, SLAC National Accelerator Laboratory, 2575 Sand Hill Road, Menlo Park, CA 94025 USA

**Keywords:** Batteries, Batteries

## Abstract

To achieve good rate capability of lithium metal anodes for high-energy-density batteries, one fundamental challenge is the slow lithium diffusion at the interface. Here we report an interpenetrated, three-dimensional lithium metal/lithium tin alloy nanocomposite foil realized by a simple calendering and folding process of lithium and tin foils, and spontaneous alloying reactions. The strong affinity between the metallic lithium and lithium tin alloy as mixed electronic and ionic conducting networks, and their abundant interfaces enable ultrafast charger diffusion across the entire electrode. We demonstrate that a lithium/lithium tin alloy foil electrode sustains stable lithium stripping/plating under 30 mA cm^−2^ and 5 mAh cm^−2^ with a very low overpotential of 20 mV for 200 cycles in a commercial carbonate electrolyte. Cycled under 6 *C* (6.6 mA cm^−2^), a 1.0 mAh cm^−2^ LiNi_0.6_Co_0.2_Mn_0.2_O_2_ electrode maintains a substantial 74% of its capacity by pairing with such anode.

## Abstract (web summary)

Sluggish lithium diffusion on the surface of Li metal anodes poses a fundamental challenge. Here the authors report a Li/Li_22_Sn_5_ alloy design to address this issue. The composite anode sustains stable Li stripping/plating cycling with a low overpotential of 20 mV under 30 mA cm^−2^ in a commercial carbonate electrolyte.

## Introduction

Lithium-ion batteries (LIBs) based on intercalation chemistry with the combination of a lithium transition metal oxide (or phosphate) cathode and a graphite anode have been widely used in consumer electronics and are making their way to electric vehicles and grids^1,2^. However, these conventional LIBs are reaching the limits regarding energy and power density^3–5^. The development of rechargeable lithium-based batteries with much higher energy and power density is of vital importance for fast expanding their applications, which certainly relies on breakthroughs in materials and electrode design^6,7^. An effective approach is to search for high-capacity anode materials with low potential against cathode materials and high lithium-ion diffusion rate to replace the most widely used graphite material, which delivers a relatively low theoretical capacity of 372 mAh g^−1^ and slow lithium-ion diffusion rate (10^−12^ and 10^−6^ cm^2^ s^−1^)^8^. Lithium metal is a holy grail anode due to its high theoretical specific capacity (3860 mAh g^−1^) and low potential (−3.040 V vs. standard hydrogen electrode). However, the practical application of lithium metal anode suffers from unsatisfactory cyclability, inferior rate capability and safety issues^9–12^. The high chemical reactivity makes lithium metal react with the liquid electrolyte to form an unstable solid-electrolyte interphase (SEI) layer. Such SEI layer breaks under considerable volume variation and repairs after the exposure of fresh lithium surface to the liquid electrolyte during cycling, leading to the continual consumption of active lithium and liquid electrolyte, and finally failure of the cell^13,14^. The slow lithium diffusion at the electrode/electrolyte interface may cause large overpotential under high current densities and therefore confine the rate capability of lithium metal anode. The infinite relative volume change of lithium metal electrode without a host material leads to the absence of the spatial control of lithium deposition and thereby the growth of lithium dendrites, and eventually causes safety concerns^9,10^.

Considerable effort has been devoted to tackling the challenges of lithium metal anodes, including electrolyte (e.g., fluorine-containing additive^15,16^, self-healing electrostatic shield^17^, fluorinated electrolyte^18^, and high salt concentration^19,20^) and interface engineering (e.g., artificial SEI^21,22^, nanoscale interfacial layer^23,24^, and lithium alloy based films^25,26^) for stabilizing the interface between the electrode and electrolyte, use of solid electrolytes for preventing dendrite growth^27,28^, and design of stable scaffolds/hosts for minimizing volume change^29–33^. These efforts effectively alleviated certain problems of lithium metal anode mainly under moderate/low current densities (e.g., <3 mA cm^−2^). However, achieving high rate capability of lithium metal anode still remains challenging^6,34^. The issues of lithium metal anode are aggravated under high current densities and high areal capacities, resulting in more severe battery failures. Recently, lithium metal structural design with high electrolyte-accessible surface area showed improved rate capability by reducing the local currents^30–32^. However, the increased contact area with electrolytes may lead to severe side reactions and reduce the lifespan of batteries. To date, there have been few significant breakthroughs that enable long-term stable cycling of lithium metal anodes at high current densities (e.g., >5 mA cm^−2^) and moderately high areal capacities (e.g., >3 mAh cm^−2^) with acceptable overpotentials in commercial carbonate electrolytes.

High rate capability of electrodes requires fast lithium-ion diffusion kinetics^34^. Lithium-rich alloy (e.g., lithium zinc alloy and lithium indium alloy) and Li_3_PO_4_ phases have high lithium-ion diffusion coefficients (10 × 10^−8^ to 10 × 10^−6^ cm^2^ s^−1^)^35–38^ and a surface layer of such phases on lithium metal has proved to be effective in improving lithium diffusion at the electrode/electrolyte interface^21,25^. However, high rate capability requires fast lithium-ion diffusion kinetics over the entire electrode, including both the surface and interior, which relies rational electrode design.

Here, we report a nanostructured lithium metal foil electrode with in situ formed three-dimensional (3D) interconnected metallic lithium and mixed electron and lithium-ion conductive lithium tin alloy (Li_22_Sn_5_) integrated networks. The 3D nanostructured metallic lithium network acts as active lithium source of the electrode. The 3D nanostructured Li_22_Sn_5_ network keeps composition and structure invariant and acts as a “pathway” for lithium diffusion and electron conduction during the stripping and plating of metallic lithium. The strong affinity between the 3D metallic Li and Li_22_Sn_5_ networks, and their abundant interfaces enable small interface impedance and therefore ultrafast lithium diffusion at these Li/Li_22_Sn_5_ interfaces. A moderate potential difference (~0.3 V) between the Li_22_Sn_5_ and metallic lithium functions as the driving force for lithium diffusion within the entire electrode. As a result, the as-achieved Li/Li_22_Sn_5_ nanocomposite delivered ultrahigh-rate capability and good stability for long-term lithium stripping/deposition cycling. Under an ultrahigh current density of 30 mA cm^−2^ and high areal capacity of 5 mAh cm^−2^, the Li/Li_22_Sn_5_ nanocomposite sustained stable electrodeposition/dissolution over 200 cycles with a very low overpotential of 20 mV in a commercial carbonate electrolyte. Furthermore, by pairing with a Li/Li_22_Sn_5_ anode, a substantial 74% of the capacity was maintained for a 1.0 mAh cm^−2^ LiNi_0.6_Co_0.2_Mn_0.2_O_2_ electrode cycled at 6 *C* (6.6 mA cm^−2^). A Li/Li_22_Sn_5_|LiFePO_4_ cell delivered a high specific capacity of 132 mAh g^−1^ at 5 *C* (4 mA cm^−2^) and showed stable and flat potential profiles with high-capacity retention of 91% for 500 cycles.

## Results

### Fabrication and characterizations of Li/Li_22_Sn_5_ nanocomposite

**Fig. 1:**
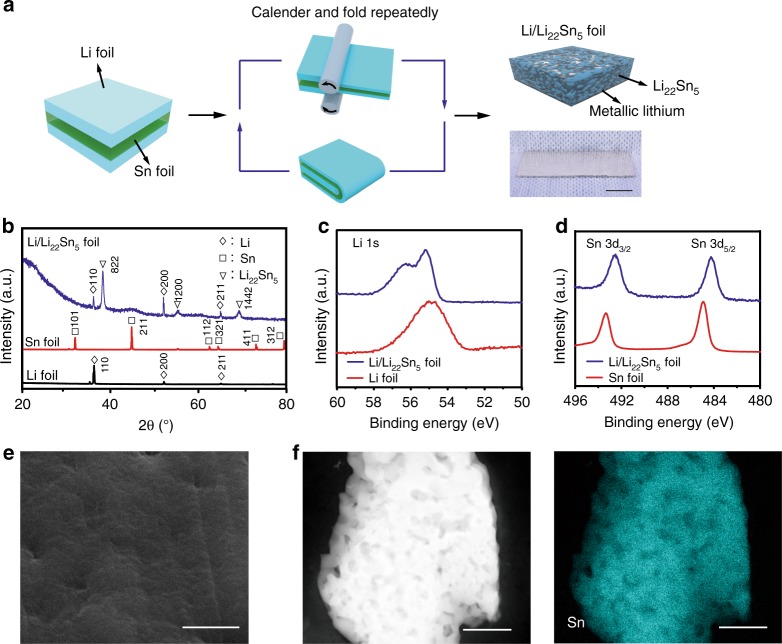
Fabrication and characterizations of the Li/Li_22_Sn_5_ nanocomposite foil. **a** Schematic of the fabrication of the Li/Li_22_Sn_5_ nanocomposite foil. Two pieces of Li metal foils and one piece of Sn foil were stacked together to form a Li–Sn–Li sandwich. Repeated calendaring and folding operations were performed for the Li–Sn–Li sandwich, producing periodically stacked metallic lithium and tin nanolayers with rich amount of Li/Sn interfaces. The metallic lithium and tin reacted spontaneously at these freshly formed interfaces, and formed a Li/Li_22_Sn_5_ nanocomposite foil. The photo of the Li/Li_22_Sn_5_ nanocomposite foil is shown in the bottom right (scale bar, 1 cm). **b** X-ray diffraction (XRD) patterns of the starting Li foil, Sn foil and the as-fabricated Li/Li_22_Sn_5_ foil. **c**, **d** High-resolution Li 1s (**c**) and Sn 3d (**d**) X-ray photoelectron spectroscopy (XPS) spectra for the pristine metallic Li foil, metallic Sn foil, and the Li/Li_22_Sn_5_ foil after surface cleaning by sputtering. **e** Top view scanning electronic microscopy (SEM) image of the Li/Li_22_Sn_5_ foil (scale bar, 1 μm). **f** Scanning transmission electron microscope (STEM) image of the Li_22_Sn_5_ and the corresponding energy dispersive X-Ray (EDX) elemental mapping image for Sn (scale bar, 500 nm). After electrochemically stripping the metallic Li away, a three-dimensional (3D) interconnected Li_22_Sn_5_ framework was observed.

The Li/Li_22_Sn_5_ nanocomposite foil was realized by a facile calendaring and folding route, and a spontaneous alloying reaction between metallic lithium and tin at room temperature in an Ar-filled glove box (Fig. 1a). During the fabrication, a tin foil was first sandwiched between two lithium foils with designed Li/Sn atomic ratio of 44/5, 88/5, or 110/5, resulting in theoretical usable Li metal capacities of 656, 1468, or 1737 mAh g^−1^ at the whole 3D nanocomposite. After 15 times of folding and calendaring operation, the thickness of each metal layer would be reduced to as low as ~5 nm in theory, producing periodically stacked metallic lithium and tin nanolayers with rich amount of Li/Sn interfaces. The metallic lithium and tin reacted spontaneously at these freshly formed interfaces and produced an interpenetrated 3D Li/Li_22_Sn_5_ nanocomposite foil [(22 + *x*)Li + 5Sn → Li_22_Sn_5_ + *x*Li(excess)], featuring 3D interconnected metallic lithium and Li_22_Sn_5_ integrated networks with strong affinity and abundant interfaces between metallic Li and Li_22_Sn_5_. Since the Li/Li_22_Sn_5_ nanocomposite foils with Li/Sn atomic ratios of 44/5 and 88/5 showed same structure and very similar electrochemical performance (discussed in the next section), we performed the detailed materials and battery characterizations using the foil with Li/Sn atomic ratio of 44/5 as an example unless otherwise stated. The Li/Li_22_Sn_5_ nanocomposite exhibits a foil structure (Fig. 1a, bottom right), similar to the initial metallic lithium and tin foils (Supplementary Fig. 1). The X-ray diffraction (XRD) results confirm the coexistence of the Li_22_Sn_5_ and metallic lithium phases in the as-achieved nanocomposite foil (Fig. 1b). The signals of metallic tin disappear in the XRD pattern of the resultant Li/Li_22_Sn_5_ nanocomposite, indicating that all the initial metallic tin has converted to Li_22_Sn_5_. X-ray photoelectron spectroscopy (XPS) analyses were performed after Ar sputtering to reveal the surface electronic state of the elemental composition. Different from a single characteristic peak at 55.0 eV in the high-resolution Li 1s spectrum for the pure lithium metal foil^39^, two distinct peaks at 55.2 eV and 56.3 eV were observed for the Li/Li_22_Sn_5_ nanocomposite, the latter of which was ascribed to the lithium element in Li_22_Sn_5_ (Fig. 1c). In the high-resolution Sn 3d spectra, the Sn 3d_5/2_ and 3d_3/2_ peaks at 484.9 eV and 493.3 eV for the pure metallic Sn foil^40^ shifted to 484.2 eV and 492.6 eV for the Li/Li_22_Sn_5_ nanocomposite, respectively, indicating the transformation of metallic Sn to Li_22_Sn_5_ (Fig. 1d). Scanning electron microscopy (SEM) investigation was further performed to investigate the morphology and structure of the Li/Li_22_Sn_5_ nanocomposite. The in situ formation of the Li_22_Sn_5_ network and its strong affinity with metallic Li make the Li_22_Sn_5_ network remain close contact with the metallic Li in the Li/Li_22_Sn_5_ composite, leading to a dense structure of the Li/Li_22_Sn_5_ composite foil (Fig. 1e). After electrochemically stripping away the metallic Li, it was observed that the Li_22_Sn_5_ has the topology of interconnected networks with the dimension of ~150 nm and numerous interconnected interspaces (Fig. 1f). Therefore, the as-fabricated Li/Li_22_Sn_5_ nanocomposite foil possesses a unique nanostructure with 3D interconnected metallic lithium and Li_22_Sn_5_ integrated networks. For a Li/Li_22_Sn_5_ composite electrode with 10 mAh cm^−2^ of metallic Li, the calculated contact area between the metallic Li and Li_22_Sn_5_ reaches 1759 or 2638 cm^2^, respectively, based on the geometry of nanowires or nanoparticles, which is three orders of magnitude higher than the accessible area to liquid electrolyte (1 cm^2^) in the conventional Li metal anode. Thus, the local current density for the metallic Li in the Li/Li_22_Sn_5_ composite electrode can be greatly reduced in comparison to that of the conventional Li metal anode when same overall current density is applied.

Such unique nanostructure allows fast lithium diffusion over the entire electrode and enables stable lithium stripping/plating cycling under high current densities without lithium dendrite growth due to its multiple advantages: First, due to the high lithium-ion diffusion coefficient, strong lithium affinity and abundant interfaces to metallic lithium, the 3D nanostructured Li_22_Sn_5_ network forms an lithium diffusion “pathway” over the entire Li/Li_22_Sn_5_ electrode. Metallic lithium can easily transport through such “pathway” back and forth. Meanwhile, the 3D nanostructured metallic lithium and Li_22_Sn_5_ networks both provide an electron “pathway” over the entire Li/Li_22_Sn_5_ electrode. Second, the potential difference (~0.3 V) between Li_22_Sn_5_ and metallic lithium can act as the driving force for lithium diffusion through such a “pathway”. These advantages enable fast lithium diffusion over the entire the Li/Li_22_Sn_5_ electrode, leading to good rate capability. Furthermore, fast lithium diffusion helps to alleviate the formation of lithium metal dendrites during cycling^41^ and thus improves the safety of rechargeable lithium metal batteries. Third, the Li_22_Sn_5_ is less reactive with the liquid electrolyte than the metallic lithium due to its higher chemical potential. Thus, less electrolyte consumption and longer cycle life can be expected. Fourth, the 3D interconnected Li_22_Sn_5_ network remains invariant in the composition and structure on cycling and is capable of working as a stable host for stripping/plating of lithium metal and thus addressing the challenge of significant volume change.

### Electrochemical performance of Li/Li_22_Sn_5_ foils

**Fig. 2:**
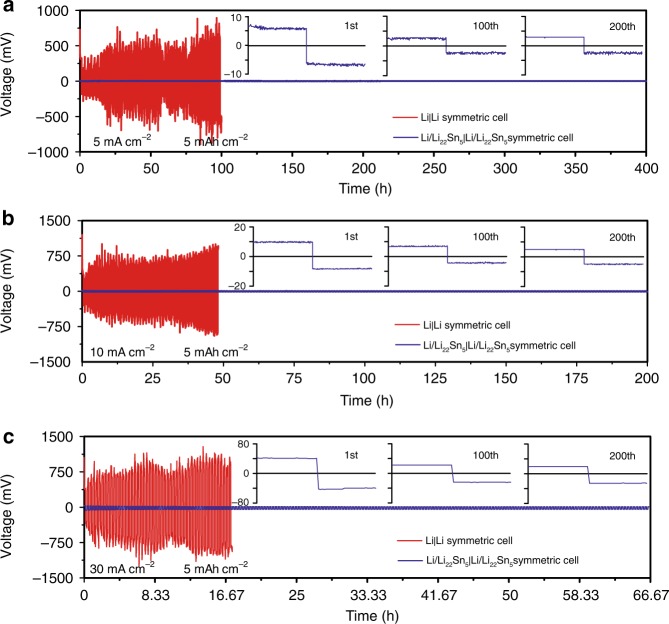
Galvanostatic lithium plating/stripping cycling and voltage profiles of the Li/Li_22_Sn_5_| Li/Li_22_Sn_5_ and Li|Li symmetric cells. **a**–**c** Lithium stripping/plating cycling of symmetric cells at 5 mA cm^−2^ (**a**), 10 mA cm^−2^ (**b**) and 30 mA cm^−2^ (**c**) with areal capacity fixed at 5 mAh cm^−2^. The insets in (**a**–**c**) are the high-resolution voltage profiles of the Li/Li_22_Sn_5_|Li/Li_22_Sn_5_ symmetric cells at the specific cycle.

To validate the advantages of the Li/Li_22_Sn_5_ electrode for high-power-density lithium metal batteries, stripping/plating measurements were performed with a practical high areal capacity of lithium (5 mAh cm^−2^) at various high current densities (5, 10, 20 and 30 mA cm^−2^) in symmetric cells using commercial carbonate-based electrolytes. Figure 2a shows the voltage profiles as a function of time for the Li/Li_22_Sn_5_|Li/Li_22_Sn_5_ symmetric cells and the Li|Li counterparts for different cycles at 5 mA cm^−2^ with fixed areal capacity of 5 mAh cm^−2^. Supplementary Fig. 2 shows the enlarged voltage profiles of Li|Li symmetric cells and Supplementary Figs. 3–5 show the enlarged voltage profiles of Li/Li_22_Sn_5_|Li/Li_22_Sn_5_ symmetric cells. For Li|Li symmetric cells cycled at 5 mA cm^−2^, a high overpotential of 0.65 V was observed at the beginning of the first cycle (Supplementary Fig. 2), corresponding to the difficulty of lithium stripping and growing beneath SEI at such a high current density. This overpotential decreased after the first cycle due to the increased real surface area caused by the growth of lithium dendrites. High overpotential was also observed at the end of each stripping/plating process after the 1st stripping process (Supplementary Fig. 2), corresponding to the usage of previously unused fresh lithium beneath the surface, which indicated the low Coulombic efficiency. Marked overpotential increase was observed after the second cycle, indicating the quick decay of the pristine lithium metal electrodes (Supplementary Fig. 2). The overpotential of Li|Li symmetric cells fluctuated wildly over each striping/plating cycle. For example, the overpotential range was 0.35–0.75 V for the stripping process at the 14th cycle (Supplementary Fig. 2). In contrast, the Li/Li_22_Sn_5_ electrode showed flat, stable, smooth voltage plateaus during lithium stripping/plating processes with a low, consistent overpotential and negligible fluctuation of ~1 mV, as well as long-term stability for 200 cycles (Fig. 2a, Supplementary Figs. 3–5). Observed from the enlarged figures (Supplementary Figs. 2 and 4), the overpotential of the Li/Li_22_Sn_5_|Li/Li_22_Sn_5_ symmetric cell at the beginning of the first cycle was two order of magnitude lower than that of the Li|Li symmetric cell (~8 mV for the Li/Li_22_Sn_5_|Li/Li_22_Sn_5_ symmetric cell vs. 0.65 V for the Li|Li symmetric cell), indicating much better charge carrier transport through the entire Li/Li_22_Sn_5_ electrode. The overpotential of the Li/Li_22_Sn_5_ electrode further decreased and remained under 5 mV after five cycles after the surface activation (Supplementary Fig. 4). Note that such low overpotential has never been reported in previous studies and it implies the significant role of the 3D nanostructured Li_22_Sn_5_ network in improving lithium diffusion over the entire electrode. Moreover, flat and stable stripping/plating plateaus with only ~ 3 mV overpotential were maintained at the 200th cycle (inset of Fig. 2a). Thus, the fast lithium diffusion kinetics of the Li/Li_22_Sn_5_ electrode enables not only low potential hysteresis, but also flat and smooth cycling plateaus with a long lifespan. Supplementary Fig. 6 compared the Nyquist plots of Li|Li and Li/Li_22_Sn_5_|Li/Li_22_Sn_5_ symmetric cells after different lithium stripping/plating cycles under 5 mA cm^−2^ with a fixed areal capacity of 5 mAh cm^−2^. The lithium foil electrode showed high interfacial resistance of ~202 Ω before cycling. It decreased to ~46 Ω after the 1st cycle due to the broken native oxide layers and increased surface area caused by the growth of lithium dendrites. Then, it increased on cycling due to the accumulation of SEI and inactive lithium^42^. In contrast, the Li/Li_22_Sn_5_ electrode had a much lower and more stable resistance during cycling. The value of interfacial resistance was ~10 Ω before cycling and remained only ~1 Ω after 10 cycles. This result supports the favorable charge carrier transport capability and stable interfacial properties of the Li/Li_22_Sn_5_ electrode.

The potential of the Li/Li_22_Sn_5_ anode for high power application was further proven by lithium stripping/plating measurements under larger current densities (10, 20 and 30 mA cm^−2^, Fig. 2b, c and Supplementary Figs. 7–15). The Li|Li symmetric cell could not sustain cycling under such high current densities, signaled by large voltage fluctuations (e.g., 0.5–1 V) (Fig. 2b, c). In sharp contrast, the Li/Li_22_Sn_5_|Li/Li_22_Sn_5_ symmetric cells exhibited significantly improved electrochemical performance. With the areal capacity fixed at 5 mAh cm^−2^, the overpotential of the Li/Li_22_Sn_5_ foil electrode increased slightly from 8 mV under 5 mA cm^−2^, 10 mV under 10 mA cm^−2^ to 20 mV under 20 mA cm^−2^ and then to 40 mV under 30 mA cm^−2^ during the initial stripping process (inset of Fig. 2 and Supplementary Fig. 11). Meanwhile, good long-term stability was achieved during the whole measurement process of 200 cycles under all the applied current densities for the Li/Li_22_Sn_5_|Li/Li_22_Sn_5_ symmetric cell (Fig. 2 and Supplementary Figs. 7–15). It is noted that 30 mA cm^−2^ is an unprecedented high current density in the measurements of lithium metal anodes, which has rarely been used, if any. The as-prepared Li/Li_22_Sn_5_ electrode exhibited unprecedented low overpotential during the lithium stripping/plating measurement under such a high current. Moreover, continuous, smooth and plat voltage plateaus were maintained for 200 cycles for the Li/Li_22_Sn_5_ electrode (Fig. 2c and Supplementary Figs. 14–16). During cycling under 30 mA cm^−2^, the overpotential of the Li/Li_22_Sn_5_|Li/Li_22_Sn_5_ symmetric cell slightly decreased from 40 mV at the 1st cycle, 30 mV at the 10th cycle, 24 mV at the 30th cycle, to 20 mV at the 50th cycle, and remained at 20 mV from 50 to 200 cycles (Fig. 2c and Supplementary Figs. 13–15). Importantly, under all applied current densities, the charge and discharge measurements of the symmetric Li/Li_22_Sn_5_|Li/Li_22_Sn_5_ cells were performed using commercial carbonate-based electrolytes with a high areal capacity of 5 mAh cm^−2^ manifesting its capability of working as high-power density and high-energy density battery anode. To the best of our knowledge, this is the best performance of lithium plating/stripping in lithium metal-based symmetric cells in consideration of applied current densities and areal capacities, compared with other Li metal studies by employing electrolyte and interface engineering, designing of stable hosts/scaffolds or using solid electrolyte^15–32^. Additionally, to investigate the effect of Li/Sn atomic ratios on the electrochemical performance of the Li/Li_22_Sn_5_ electrodes, we also tested the symmetric cells with higher Li/Sn atomic ratios of 88/5 and 110/5, in addition to 44/5 in the above discussion. With fixed areal capacity of 5 mAh cm^−2^ at 10 mA cm^−2^, the Li/Li_22_Sn_5_ electrode with a Li/Sn atomic ratio of 88/5 showed low initial overpotential (14 mV for the initial stripping process and ~10 mV on cycling) and stable cycling for 200 cycles (Supplementary Figs. 16–18), which was similar to the electrode with a Li/Sn atomic ratio of 44/5 (Supplementary Figs. 7–9). However, the electrode with a Li/Sn atomic ratio of 110/5 exhibited much larger overpotential than the counterparts with low Li/Sn ratios and decayed within 10 cycles (Supplementary Fig. 19), since the 3D mixed conducting Li_22_Sn_5_ network could not form in the electrode due to the low content of Sn. Moreover, the lithium in the Li/Li_22_Sn_5_ electrode could be fully extracted after 200 stripping/plating cycles at 30 mA cm^−2^ and 5 mAh cm^−2^ (Supplementary Fig. 21). The voltage rapidly reached 1 V after the exhaustion of all the stored lithium during the lithium extraction process. The sharp increase in voltage after the full stripping of metallic Li for the cycled electrode verified that short did not take place for the Li/Li_22_Sn_5_|Li/Li_22_Sn_5_ cells after 200 cycles cycled at 30 mA cm^−2^ and 5 mAh cm^−2^, which meets the demand for high-power-density applications, such as drone.

**Fig. 3:**
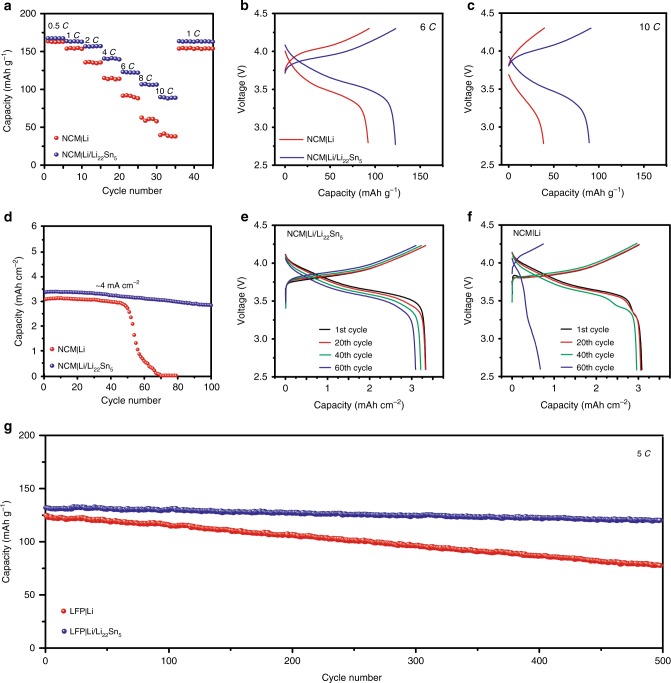
Electrochemical performance of LiNi_0.6_Co_0.2_Mn_0.2_O_2_ (NCM)|Li/Li_22_Sn_5_ and LiFePO_4_ (LFP)| Li/Li_22_Sn_5_ cells. **a** Rate capability of the NCM|Li/Li_22_Sn_5_ and NCM|Li cells with NCM loading of ~6.5 mg cm^−2^ at various rates from 0.5 to 10 *C*. **b**, **c** Voltage vs. capacity profile comparison of the NCM|Li/Li_22_Sn_5_ and NCM|Li cells at rates of 6 *C* (**b**) and 10 *C* (**c**). **d** Capacity vs. cycle number profiles of the NCM|Li/Li_22_Sn_5_ and NCM|Li cells with high NCM mass loading (~23.7 mg cm^−2^) and low areal capacity ratio of negative to positive electrodes (N/P ratio, 3.75). **e**, **f** Voltage vs. capacity profiles of the the NCM|Li/Li_22_Sn_5_ (**e**) and NCM|Li (**f**) cells with high NCM mass loading and low N/P ratio for various cycles. **g** Capacity vs. cycle number profiles of the LFP|Li/Li_22_Sn_5_ and LFP|Li cells with LFP mass loading of ~5 mg cm^−2^ at 5 *C* for 500 cycles.

To validate all these superiorities of the Li/Li_22_Sn_5_ electrode for high-power and high-energy density lithium metal batteries, full cells were constructed by using Li/Li_22_Sn_5_ anodes paired with LiNi_0.6_Co_0.2_Mn_0.2_O_2_ (NCM) and LiFePO_4_ (LFP) cathodes. The electrochemical measurement of NCM|Li/Li_22_Sn_5_ and NCM|Li cells was performed at increasing current rates based on a theoretical specific capacity of 170 mAh g^−1^ for NCM and ~6.5 mg cm^−2^ active mass loading (Fig. 3a–c). The NCM|Li/Li_22_Sn_5_ cells delivered capacities of 167 mAh g^−1^ at 0.5 *C*, 163 mAh g^−1^ at 1 *C*, 157 mAh g^−1^ at 2 *C*, 141 mAh g^−1^ at 4 *C*, 123 mAh g^−1^ at 6 *C*, 107 mAh g^−1^ at 8 *C* and 90 mAh g^−1^ at 10 *C* (Fig. 3a). With a capacity of 123 mAh g^−1^ at a high current density of 6 *C* (6.6 mA cm^−2^), such a NCM|Li/Li_22_Sn_5_ cell can fill up 74% of its capacity within 10 mins, suggesting the good rate capability of the Li/Li_22_Sn_5_ electrode. Similar rate capacities were achieved on five NCM|Li/Li_22_Sn_5_ cells, showing the repeatability (Supplementary Fig. 22). In comparison, the NCM|Li cell gave much lower capacities, especially at high rates. For example, the capacities of NCM|Li cell were 163 mAh g^−1^ at 0.5 *C*, 136 mAh g^−1^ at 2 *C*, 92 mAh g^−1^ at 6* C* and 40 mAh g^−1^ at 10 *C* (Fig. 3a). Moreover, smaller voltage hysteresis and higher discharge voltage were observed for the NCM cell using Li/Li_22_Sn_5_ anode compared to that using pristine Li metal anode at 6 *C* (Fig. 3b, c), in accordance with the voltage profiles of the symmetric cells (Fig. 2, Supplementary Figs. 2–5, and Supplementary Figs. 7–15). Impressively, Li_4_Ti_5_O_12_ (LTO)|Li/Li_22_Sn_5_ cells exhibited a stable discharge capacity of 1 mAh cm^−2^ at 33.1 mA cm^−2^ (15 *C*), suggesting the good stability of the Li/Li_22_Sn_5_ electrode at ultrahigh current density (Supplementary Fig. 23). Full cells with high cathode mass loading (NCM, ~23.7 mg cm^−2^) and low areal capacity ratio of negative to positive electrodes (N/P ratio, 3.75) were further built and their electrochemical performance was investigated. A NCM|Li/Li_22_Sn_5_ full cell delivered an initial capacity of 3.33 mAh cm^−2^ for the 1st cycle and 2.80 mAh cm^−2^ for the 100th cycle at 4 mA cm^−2^, with a capacity retention of 84 % (Fig. 3d, e). In contrast, a NCM|Li full cell with the same loading of electrodes displayed a slightly lower capacity of 3.04 mAh cm^−2^ for the 1st cycle and sustained a much smaller number of cycles (50 cycles, Fig. 3d, f).

Due to the good long-term cycling stability of LFP, LFP|Li/Li_22_Sn_5_ cells were constructed to investigate the cycling stability of the Li/Li_22_Sn_5_ electrode. With typical LFP mass loading of ~5 mg cm^−2^, the LFP|Li/Li_22_Sn_5_ cell offered a high capacity of 132 mAh g^−1^ at a high rate of 5 *C* (4 mA cm^−2^) for the 1st cycle and 120 mAh g^−1^ for the 500th cycle, delivering high-capacity retention of 91%. Also, the capacity vs. cycle number profile of the LFP|Li/Li_22_Sn_5_ cell showed little fluctuation during cycling (Fig. 3g). As a contrast, LFP|Li cell displayed much lower capacity and obviously capacity decay on cycling (Fig. 3g). Its capacity was only 78 mAh g^−1^ for the 500th cycle with its capacity retention of only 62%. An in-depth comparison of voltage profiles for LFP cells paired with Li/Li_22_Sn_5_ anode and Li metal anode was shown in Supplementary Fig. 21. As we prolonged the cycle number, the voltage profiles of the LFP|Li/Li_22_Sn_5_ cell remained stable. Thus, the Li/Li_22_Sn_5_ foil electrode is able to survive stable and extensive cycling. In contrast, the LFP|Li cell showed an obvious increase in voltage hysteresis on cycling. Much smaller overpotential were observed for the LFP|Li/Li_22_Sn_5_ cell at 1st, 150th, and 300th cycle compared to LFP|Li cell (Supplementary Fig. 24). The remarkable electrochemical performance of the NCM|Li/Li_22_Sn_5_ and LFP|Li/Li_22_Sn_5_ cells further verifies the potential application of the Li/Li_22_Sn_5_ anode in industrialized lithium metal batteries.

Li_4_Ti_5_O_12_ (LTO) can be an ideal Li reservoir to evaluate the Coulombic efficiency of a Li metal anode since it has near 100% Coulombic efficiency and does not provide Li during cycling. 10 mAh cm^−2^ Li/Li_22_Sn_5_ electrode and pristine Li metal electrode were paired with ~2.8 mAh cm^−2^ of LTO at 1.4 mA cm^−2^ (0.5 *C*) in a commercial carbonate electrolyte. The capacity of the LTO|Li cell started to quickly decay at the 30th cycle, showing a Coulombic efficiency of 91.2%, and the LTO|Li cell lost all its capacity after 60 cycles (Supplementary Fig. 25a). In contrast, the capacity of the LTO|Li/Li_22_Sn_5_ cell remained constant (~155 mAh g^−1^) for 75 cycles, giving a higher Coulombic efficiency of 96.5% (Supplementary Fig. 25a). Meanwhile, much smaller voltage hysteresis was also observed for the LTO|Li/Li_22_Sn_5_ cell in comparison to the LTO|Li cell during cycling (Supplementary Fig. 25b, c).

### Characterizations of Li stripping/plating

**Fig. 4:**
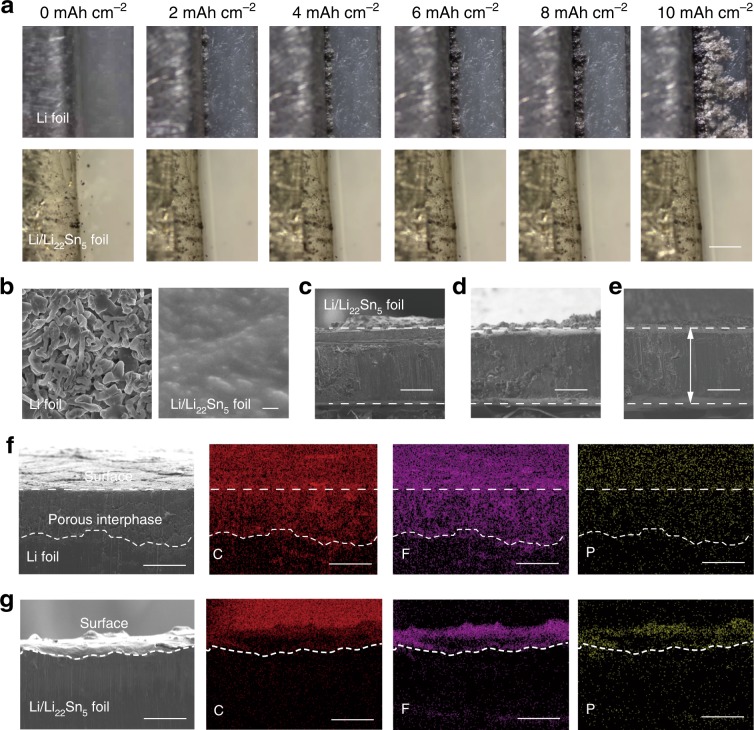
Optical microscopy and SEM investigation of Li/Li_22_Sn_5_ electrode. **a** In situ optical microscopy investigation of the interfaces between the electrolyte and electrode for the Li|Li (up) and Li/Li_22_Sn_5_|Li/Li_22_Sn_5_ (down) symmetric cells during lithium plating process (1 mA cm^−2^, 10 mAh cm^−2^) (scale bar, 300 μm). **b** Top view SEM images of Li foil (left) and Li/Li_22_Sn_5_ foil (right) after a stripping/plating cycle with 5 mAh cm^−2^ of lithium under 5 mA cm^−2^ (scale bar, 1 μm). **c**–**e** Cross-sectional SEM images of a Li/Li_22_Sn_5_ foil before electrochemical lithium stripping/plating cycling (**c**), after stripping 10 mAh cm^−2^ of lithium under 5 mA cm^−2^ (**d**), and after a stripping/plating cycle with 10 mAh cm^−2^ of lithium under 5 mA cm^−2^ (**e**) (scale bar, 200 μm). **f**, **g** Cross-sectional SEM images and the corresponding EDX mapping images of a Li foil (**f**) and a Li/Li_22_Sn_5_ foil (**g**) after 50 stripping/plating cycles with 5 mAh cm^−2^ of lithium under 5 mA cm^−2^ (scale bar, 50 μm).

In situ optical microscopy and ex situ SEM characterizations were performed to investigate the lithium stripping and plating behavior of the Li/Li_22_Sn_5_ nanocomposite foil. To directly monitor the lithium plating process, transparent Li|Li and Li/Li_22_Sn_5_|Li/Li_22_Sn_5_ symmetric cells were assembled and performed at 1 mA cm^−2^ under an optical microscope (Fig. 4a). The formation process of lithium dendrites on the bare lithium foil was clearly observed. These dendrites grew heterogeneously and some of them extended to more than 300 μm in distance in the observed area after 10 h. In contrast, the situation was very different for the Li/Li_22_Sn_5_ electrode. There is no dendrite formation on the electrode surface even after 10 h’ plating of lithium metal at 1 mA cm^−2^. Slight movement of the electrode/electrolyte interface was observed due to the uniform lithium plating. Figure 4b compared top-view SEM images of the pristine Li metal electrode and Li/Li_22_Sn_5_ electrode after a stripping/plating cycle with 5 mAh cm^−2^ of lithium under 5 mA cm^−2^. A large amount of lithium dendrites was observed on the lithium foil surface (Fig. 4b left), while a smooth surface was maintained for the Li/Li_22_Sn_5_ foil due to the fast lithium diffusion, and uniform stripping and plating of lithium metal (Fig. 4b right). Therefore, the Li/Li_22_Sn_5_ nanocomposite foil is capable of working stably without dendrite growth and structural change. Previously, successful examples of surface modification and engineering were shown to suppress the growth of lithium dendrites^21–26^. In this work, we alleviate the formation of lithium metal dendrites and improve the safety of rechargeable lithium metal batteries through improving the lithium diffusion over the entire electrode by the in situ formed 3D nanostructured mixed conducting Li_22_Sn_5_ framework.

Volume stability of the Li/Li_22_Sn_5_ foil was investigated through thickness measurement after lithium stripping/plating cycles. It was observed that the thickness of the Li/Li_22_Sn_5_ foil did not change obviously before electrochemical lithium stripping (Fig. 4c), after stripping 10 mAh cm^−2^ of lithium under 5 mA cm^−2^ (Fig. 4d), and after a stripping/plating cycle with 10 mAh cm^−2^ of lithium under 5 mA cm^−2^ (Fig. 4e). The negligible thickness change for the Li/Li_22_Sn_5_ electrode confirms that the Li_22_Sn_5_ framework works effectively as a stable skeleton, which guarantees the uniform lithium plating and stripping, and avoids the growth of lithium dendrites on the electrode surface during cycling. Moreover, the top-view SEM images do not show obvious pore structure or the exposure of the Li_22_Sn_5_ matrix for the Li/Li_22_Sn_5_ foil after stripping 5 mAh cm^−2^ of lithium under 5 mA cm^−2^ (Supplementary Fig. 28). This result supports that the lithium stripping processes take place over the entire Li/Li_22_Sn_5_ electrode, not only the surface, because of the lithium diffusion and electron transfer “pathway” provided by the 3D Li_22_Sn_5_ network and the driving force for lithium diffusion provided by the potential difference between Li_22_Sn_5_ and metallic lithium. The 3D Li_22_Sn_5_ nanostructured network remains close contact with the metallic lithium due to the good lithium affinity^43^, and keeps lithium ion and electron conductive on the whole lithium stripping/plating cycling. Meanwhile, the 3D metallic lithium nanostructured network provides good electron conductivity over the entire Li/Li_22_Sn_5_ electrode.

**Fig. 5:**
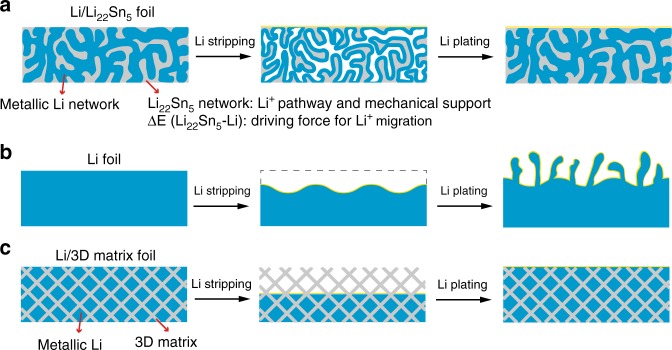
Schematic of lithium stripping/plating process of Li/Li_22_Sn_5_ nanocomposite foil, lithium foil, and Li metal with 3D matrix (Li/3D matrix). In the Li/Li_22_Sn_5_ foil, the in situ formed nanostructured 3D Li_22_Sn_5_ and metallic lithium network interconnect with each to form an integrated structure with abundant Li/Li_22_Sn_5_ interfaces with low interface impedance due to the good affinity between Li_22_Sn_5_ and metallic lithium. The 3D Li_22_Sn_5_ network works as a lithium diffusion and electron transfer “pathway” and its potential difference with metallic lithium provides the driving force for lithium diffusion. Thus, the stripping and plating of metallic lithium metal takes place over the entire Li/Li_22_Sn_5_ electrode (**a**). The pristine lithium metal foil electrode shows inhomogeneous lithium stripping and plating behavior and dendrite growth takes place during cycling (**b**). For the Li metal anode with “non-lithium ion conductive” 3D matrixes, the dissolution of lithium metal starts on the top or surface of the composite electrode during the lithium stripping process. Metallic lithium deposits back into the 3D porous structure during the lithium plating process (**c**).

To verify the different lithium stripping and plating behaviors of the bare Li metal and Li/Li_22_Sn_5_ electrodes, the structure of the two electrodes were investigated after 50 cycles of lithium stripping/plating at 5 mA cm^−2^ and 5 mAh cm^−2^. The cross-section SEM image of the bare Li metal electrode shows a porous and loose interphase layer with the thickness of ~100 μm above the dense pristine Li metal layer and the corresponding energy dispersive X-Ray (EDX) elemental mapping images exhibit strong signals of C, F, and P elements on the surface layer and weak signals on the bottom layer (Fig. 4f). This result indicates that the SEI layer and electrolyte (LiPF_6_ based carbonate liquid electrolyte) extend within the structure as cycling takes place. The formation of this loose interphase consumes active lithium and electrolyte, suggesting the serious corrosion of the bulk Li metal electrode. In contrast, the Li/Li_22_Sn_5_ electrode preserves its dense structure and the signals of C, F, and P elements are very weak over the entire cross section (Fig. 4g). Therefore, the SEI layer of the Li/Li_22_Sn_5_ electrode mainly remains fixed in place on top of the electrode and the Li/Li_22_Sn_5_ electrode excludes the electrolyte from within the electrode during cycling. The schematic of the lithium stripping and plating process of the Li/Li_22_Sn_5_ electrode is shown in Fig. 5a. Such lithium stripping and plating process differs from the bare lithium metal foil electrode, which shows inhomogeneous stripping and plating of lithium (Fig. 5b). Also, our design here is significantly different from previous reports on lithium metal anodes using a “non-lithium ion conductive” 3D matrixes (e.g., reduced graphene oxide^30^, carbon nanofiber^44^, and nickel foam^45^). Although the volume change at the electrode level would be reduced by using these mechanical supports, side reactions between the electrolyte and electrode would be enhanced due to the movement of electrode/electrolyte interface (Fig. 5c). Meanwhile, these electrodes with “non-lithium ion conductive” 3D matrixes cannot sustain cycling under high current densities, since the lithium diffusion within these electrodes is still limited.

## Discussion

In this work, we demonstrated that, a unique nanostructured lithium metal electrode with 3D interconnected metallic lithium and Li_22_Sn_5_ integrated networks, generated by the spontaneous reaction between periodically stacked nanolayers of metallic lithium and tin, enabled stable lithium stripping/plating cycling under ultrahigh current densities. Due to the high lithium-ion diffusion coefficient, good lithium affinity and moderately potential difference between Li_22_Sn_5_ and metallic lithium, the 3D nanostructured Li_22_Sn_5_ network, where metallic lithium closely contacts, provides a pathway and driving force for fast lithium diffusion over the entire Li/Li_22_Sn_5_ electrode. Also, such 3D nanostructured Li_22_Sn_5_ network enables fast electron transfer and functions as a stable host to minimize the volume change during lithium stripping and plating processes. It is worthwhile that our design here is significantly different from surface engineering with lithium conductive layer on pristine lithium foil or 3D inert mechanical support in previous studies. Lithium diffuses rapidly over the whole Li/Li_22_Sn_5_ electrode through the rich amount of Li/Li_22_Sn_5_ interfaces and 3D Li_22_Sn_5_ network. The Li/Li_22_Sn_5_|Li/Li_22_Sn_5_ symmetric cell showed stable lithium stripping/plating cycling under 30 mA cm^−2^ at 5 mAh cm^−2^ for 200 cycles. A substantial 74% of the capacity was maintained for a 1.0 mAh cm^−2^ NCM electrode cycled at 6 *C* (6.6 mA cm^−2^) by pairing such a Li/Li_22_Sn_5_ anode. High capacity retention of 91% and stable potential profiles were achieved for an LFP|Li/Li_22_Sn_5_ cell for 500 cycles at 5 *C* (4 mA cm^−2^). These results suggest the potential application of Li/Li_22_Sn_5_ nanostructured electrode in lithium metal batteries with high power and long lifespan.

## Methods

### Materials synthesis

The fabrication of the Li/Li_22_Sn_5_ foil was realized using a repeated folding and calendaring method, and a spontaneous reaction between metallic lithium and tin. A tin foil and two lithium foils with same size and designed ratio of Li/Sn were first stacked to form a Li–Sn–Li “sandwich” and pressed together by mechanical rolling using a roll squeezer in an Ar-filled glove box. The overall thickness of the produced Li–Sn–Li foil was 0.5 mm, which could be changed by tuning the spacing between the two rollers of the roll squeezer. The Li–Sn–Li “sandwich” was folded and rolled repeatedly, which led to the gradual increase in the number and reduction in the thickness of each metal layer. Such process produced rich amount of fresh Li/Sn interfaces, where the metallic lithium and tin reacted spontaneously, and formed a 3D Li_22_Sn_5_ framework with excess lithium evenly embedded in [(22 + *x*)Li + 5Sn → Li_22_Sn_5_ _+_ *x*Li (excess)]. The as-achieved Li/Li_22_Sn_5_ foil was cut into 12-mm-diameter electrodes for electrochemical measurements.

### Characterization

The morphology, microstructure, and component of the Li/Li_22_Sn_5_ foil were investigated using XRD (PANalytical B.V., Holland), field-emission scanning electrode microscopy (FESEM, Sirion 200), X-ray photoelectron spectroscopy (XPS, VG Multilab 2000) and transmission electron microscopy (TEM, Talos F200X). Before the XRD measurement, samples were loaded on a glass slide and covered with Kapton tape in the Ar-filled glove box to avoid the reactions between the samples and ambient air. Samples for SEM, XPS, and TEM measurements were sealed in the Ar-filled glove box before being transferred into the chamber of the equipment. To observe the Li_22_Sn_5_ framework of the Li/Li_22_Sn_5_ foil, metallic Li was electrochemically stripped away using a Li/Li_22_Sn_5_|Li cell configuration. The sample for characterization was rinsed by dimethyl carbonate. In situ optical microscopy (Olympus, BX53M) observations of lithium metal electrodeposition on the Li/Li_22_Sn_5_ and the pristine Li foil substrates were carried out using a side-by-side-type cell (EC-CELL, ECC-Opto-SBS). The Li metal counter and reference electrodes and GFF separator were assembled in an argon-filled glove box with less than 1 ppm of oxygen and moisture. The monitoring of the Li metal was performed by a BioLogic SP-300.

### Electrochemical measurements

2032 coin-type cells were assembled in an Ar-filled glove box for electrochemical measurements. The electrolyte was 1 M lithium hexafluorophosphate (LiPF_6_) in 1:1:1 ethylene carbonate (EC)/propylene carbonate (PC)/diethyl carbonate (DEC) with 10% fluoroethylene carbonate (FEC) and 1% vinylene carbonate (VC). Celgard 2300 (19 μm, PP/PE/PP) was used as the separator. Battery performance was investigated in a galvanostatic mode at various current densities using a LAND battery tester. The electrochemical impedance spectroscopy (EIS) measurement was performed on a Biologic VMP3 system. The NCM and LFP electrodes were fabricated with 80% active materials, 10% polyvinylidene fluoride (PVDF) and 10% carbon black. The active mass loadings of NCM and LFP electrodes were ~6.5 and ~5.0 mg cm^−2^, respectively. The current rates for NCM and LFP are based on their practical specific capacities of 170 mAh g^−1^ and 160 mAh g^−1^, respectively. For full cell measurement with high mass loading, the active mass loading of NCM electrode was ~23.7 mg cm^−2^.

## Supplementary information

Supplementary Information

Description of Additional Supplementary Files

Supplementary Movie 1

Supplementary Movie 2

## Data Availability

The data that support the plots within this paper and other finding of this study are available from the corresponding author upon reasonable request.
