# Supplementary material for: Mechanical rolling formation of interpenetrated lithium metal/lithium tin alloy foil for ultrahigh-rate battery anode
Source: Nat Commun. 2020 Feb 11;11:829. doi: 10.1038/s41467-020-14550-3 (PMC7012843; doi:10.1038/s41467-020-14550-3)
Supplement: Supplementary file 1 — Supplementary Information [file 41467_2020_14550_MOESM1_ESM.pdf]

Mechanical rolling formation of interpenetrated lithium metal/lithium tin alloy foil for ultrahigh-rate battery anode

Wan et al.

Supplementary Information include:

Supplementary Figures 1 to 31

Supplementary Notes 1 to 5

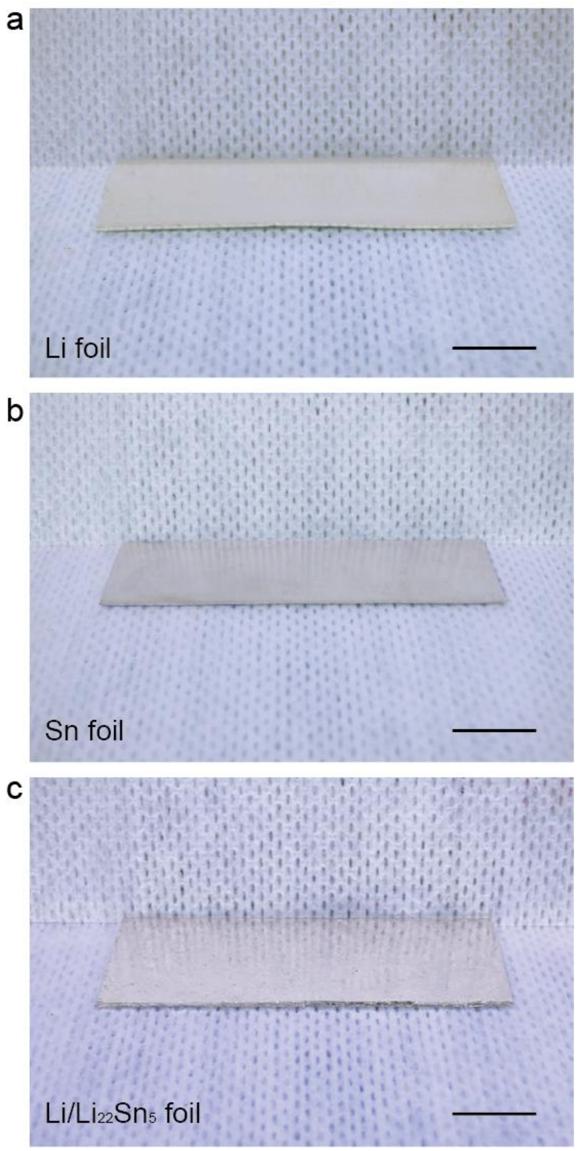

Supplementary Figure 1 | Digital images of a metallic Li foil, a metallic Sn foil and a Li/$Li_{22}Sn_5$ nanocomposite foil (scale bar, 1 cm).

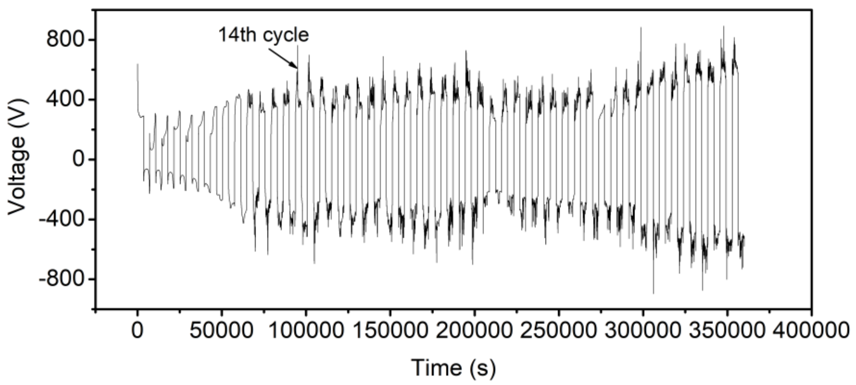

Supplementary Figure 2 | The enlarged voltage profiles of Li|Li symmetric cells cycled at 5 mA cm^{-2} with fixed areal capacity of 5 mAh cm^{-2}.

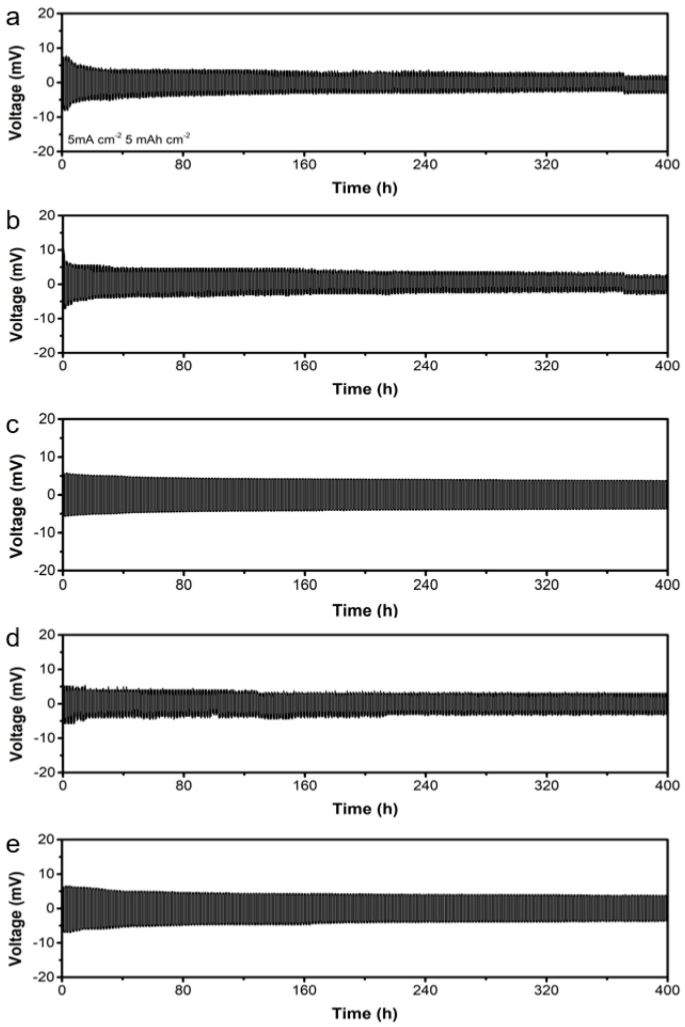

Supplementary Figure 3 | The enlarged voltage profiles of 5 Li/$Li_{22}Sn_5$|Li/$Li_{22}Sn_5$ symmetric cells cycled at 5 mA cm^{-2} with fixed areal capacity of 5 mAh cm^{-2}.

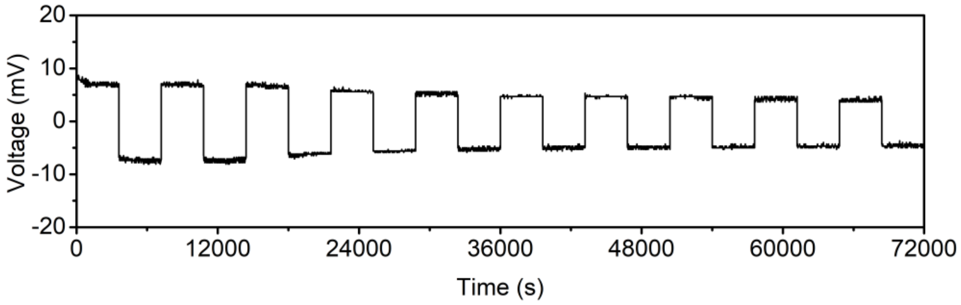

Supplementary Figure 4 | The enlarged voltage profiles of Li/$Li_{22}Sn_5$|Li/$Li_{22}Sn_5$ symmetric cells from 1 to 10 cycles at 5 mA cm^{-2} with fixed areal capacity of 5 mAh cm^{-2} (corresponding to Supplementary Fig. 3a).

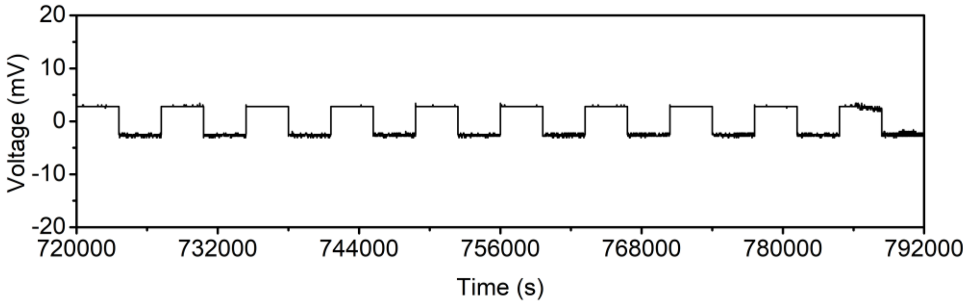

Supplementary Figure 5 | The enlarged voltage profiles of Li/$Li_{22}Sn_5$|Li/$Li_{22}Sn_5$ symmetric cells from 100 to 110 cycles at 5 mA cm^{-2} with fixed areal capacity of 5 mAh cm^{-2} (corresponding to Supplementary Fig. 3a).

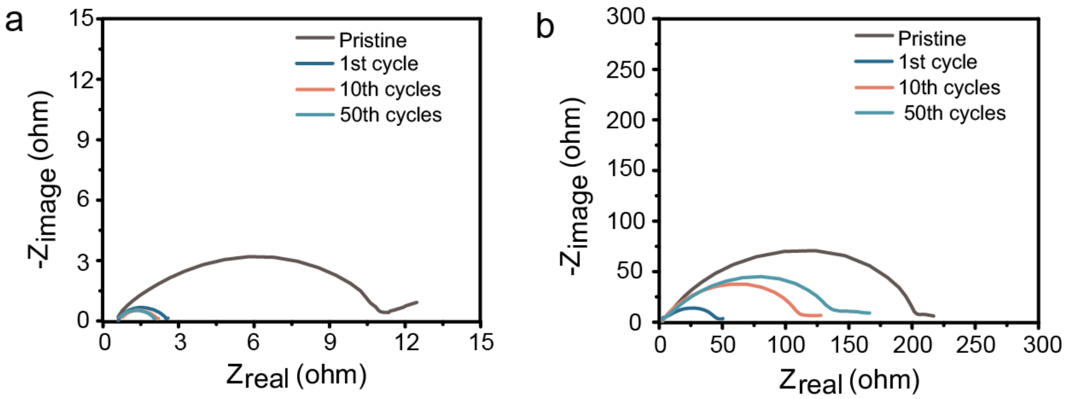

Supplementary Figure 6 | Nyquist plots of Li/$Li_{22}Sn_5$|Li/$Li_{22}Sn_5$ and Li|Li symmetric cells after different lithium stripping/plating cycles under 5 mA cm^{-2} with fixed areal capacity of 5 mAh cm^{-2}. The Li/$Li_{22}Sn_5$ electrode showed a much lower and more stable resistance than the pristine Li electrode during cycling. The value of resistance was ~0.7 ohms cm^{-2} for the separator and electrolyte.

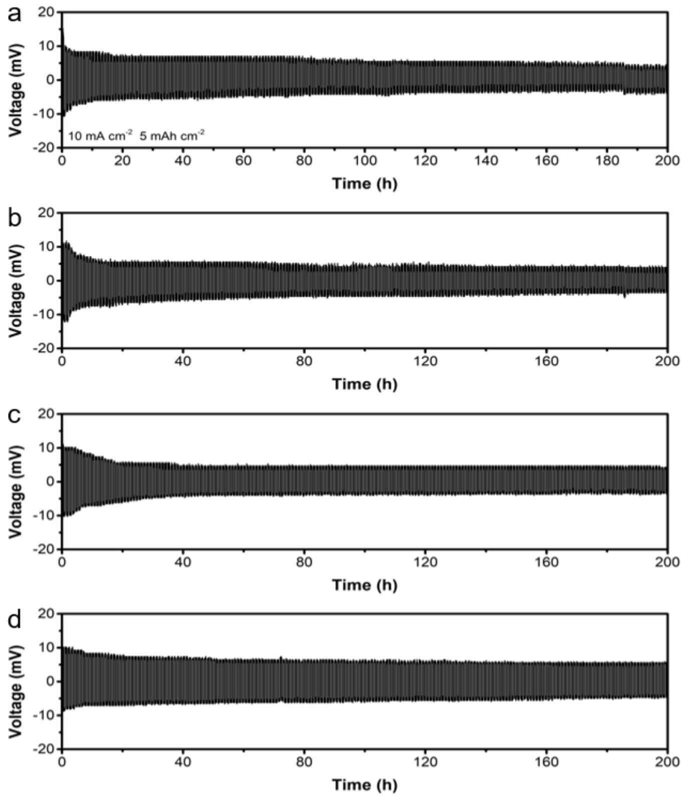

Supplementary Figure 7 | The enlarged voltage profiles of 4 Li/$Li_{22}Sn_5$|Li/$Li_{22}Sn_5$ symmetric cells cycled at 10 mA cm^{-2} with fixed areal capacity of 5 mAh cm^{-2}.

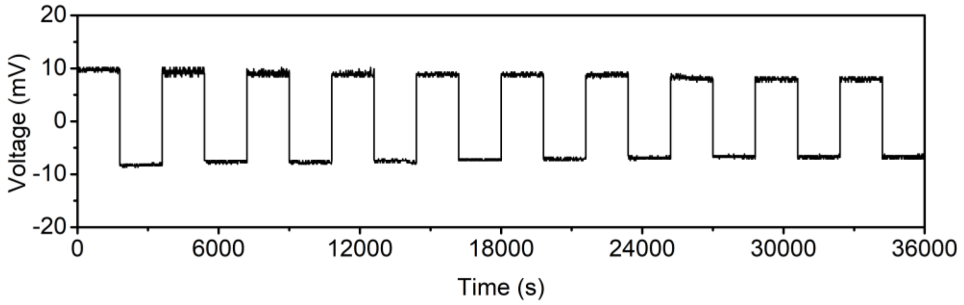

Supplementary Figure 8 | The enlarged voltage profiles of Li/$Li_{22}Sn_5$|Li/$Li_{22}Sn_5$ symmetric cells from 1 to 10 cycles at 10 mA cm^{-2} with fixed areal capacity of 5 mAh cm^{-2} (corresponding to Supplementary Fig. 7a).

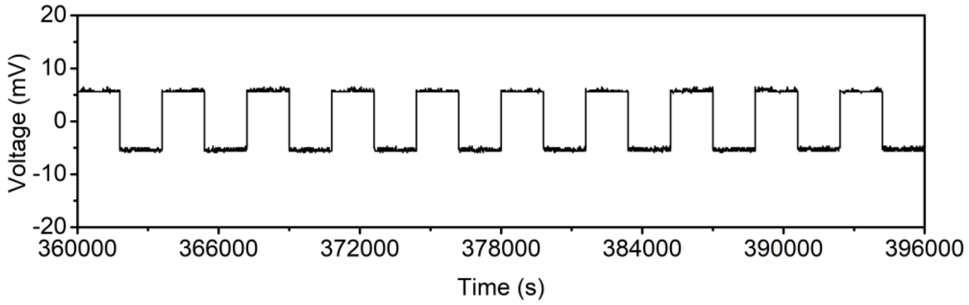

Supplementary Figure 9 | The enlarged voltage profiles of Li/$Li_{22}Sn_5$|Li/$Li_{22}Sn_5$ symmetric cells from 100 to 110 cycles at 10 mA cm^{-2} with fixed areal capacity of 5 mAh cm^{-2} (corresponding to Supplementary Fig. 7a).

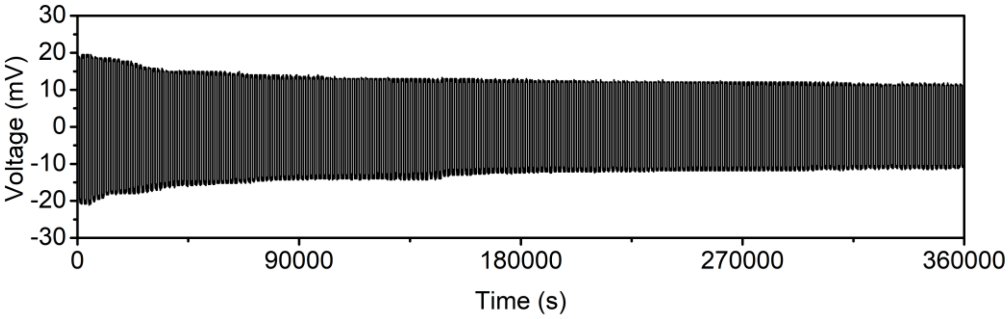

Supplementary Figure 10 | The enlarged voltage profiles of Li/$Li_{22}Sn_5$|Li/$Li_{22}Sn_5$ symmetric cells cycled at 20 mA cm^{-2} with fixed areal capacity of 5 mAh cm^{-2}.

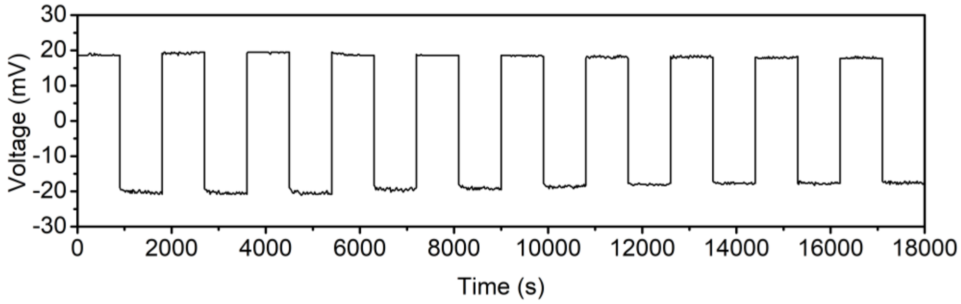

Supplementary Figure 11 | The enlarged voltage profiles of Li/$Li_{22}Sn_5$|Li/$Li_{22}Sn_5$ symmetric cells from 1 to 10 cycles at 20 mA cm^{-2} with fixed areal capacity of 5 mAh cm^{-2}.

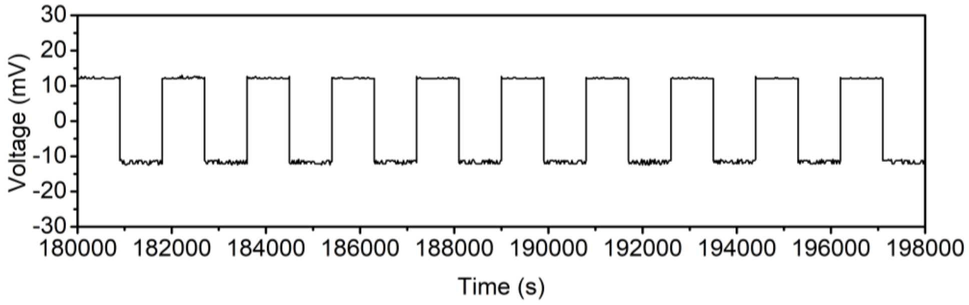

Supplementary Figure 12 | The enlarged voltage profiles of Li/$Li_{22}Sn_5$|Li/$Li_{22}Sn_5$ symmetric cells from 100 to 110 cycles at 20 mA cm^{-2} with fixed areal capacity of 5 mAh cm^{-2}.

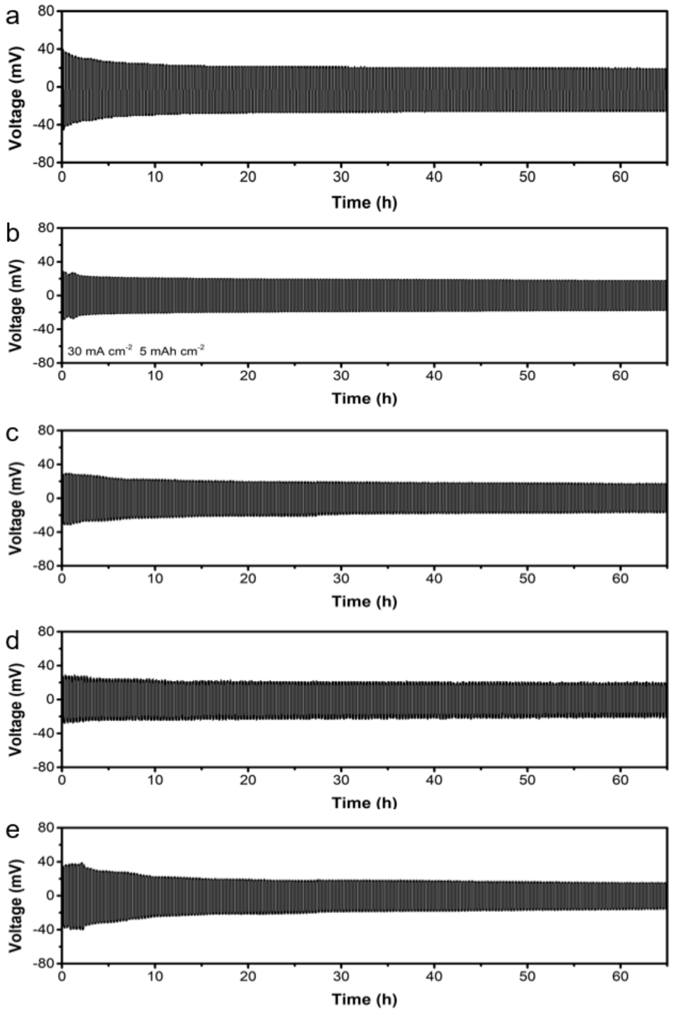

Supplementary Figure 13 | The enlarged voltage profiles of 5 Li/$Li_{22}Sn_5$|Li/$Li_{22}Sn_5$ symmetric cells cycled at 30 mA cm^{-2} with fixed areal capacity of 5 mAh cm^{-2}.

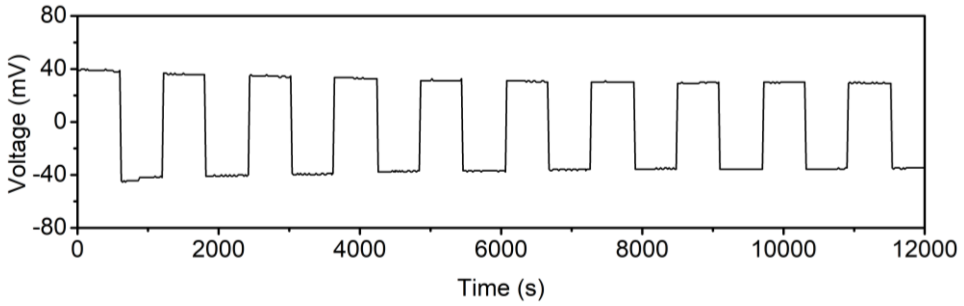

Supplementary Figure 14 | The enlarged voltage profiles of Li/$Li_{22}Sn_5$|Li/$Li_{22}Sn_5$ symmetric cells from 1 to 10 cycles at 30 mA cm^{-2} with fixed areal capacity of 5 mAh cm^{-2} (corresponding to Supplementary Fig. 13a).

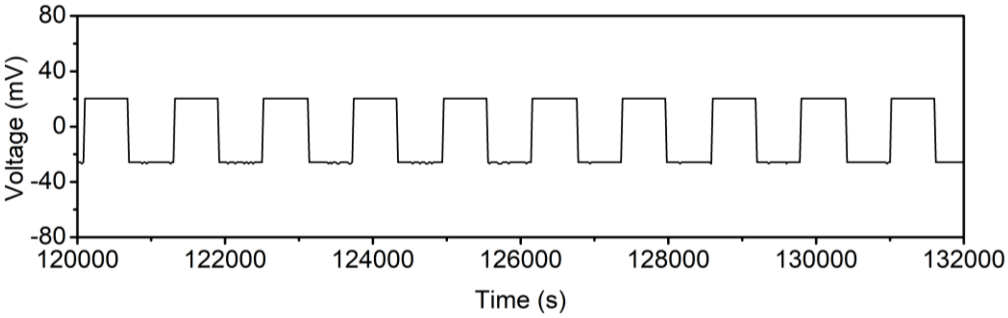

Supplementary Figure 15 | The enlarged voltage profiles of Li/$Li_{22}Sn_5$|Li/$Li_{22}Sn_5$ symmetric cells from 100 to 110 cycles under 30 mA cm^{-2} with fixed areal capacity of 5 mAh cm^{-2} (corresponding to Supplementary Fig. 13a).

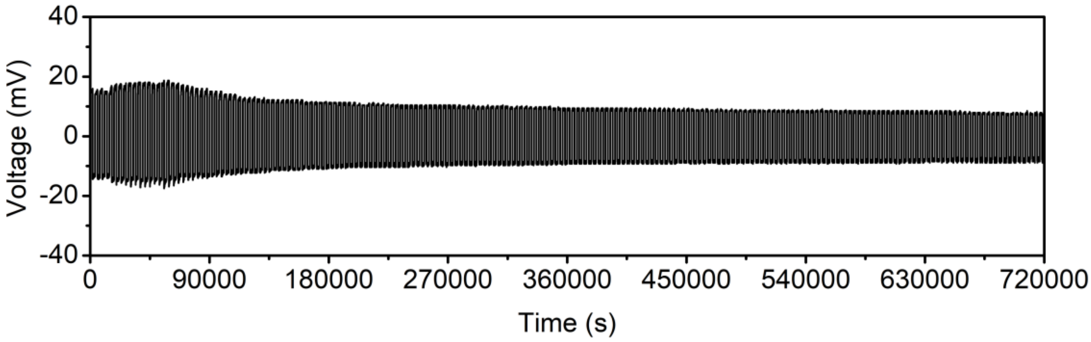

Supplementary Figure 16 | The voltage profiles of Li/$Li_{22}Sn_5$|Li/$Li_{22}Sn_5$ symmetric cells with a Li/Sn atomic ratio of 88/5 in the electrode cycled at 10 mA cm^{-2} with fixed areal capacity of 5 mAh cm^{-2}.

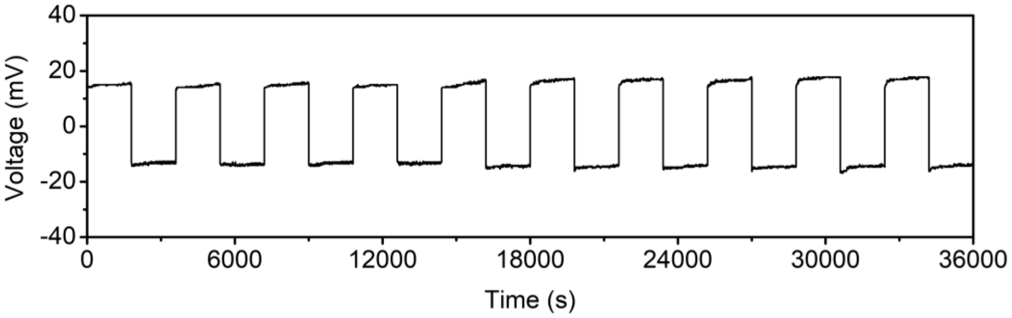

Supplementary Figure 17 | The enlarged voltage profiles of Li/$Li_{22}Sn_5$|Li/$Li_{22}Sn_5$ symmetric cells with a Li/Sn atomic ratio of 88/5 in the electrode from 1 to 10 cycles at 10 mA cm^{-2} with fixed areal capacity of 5 mAh cm^{-2}.

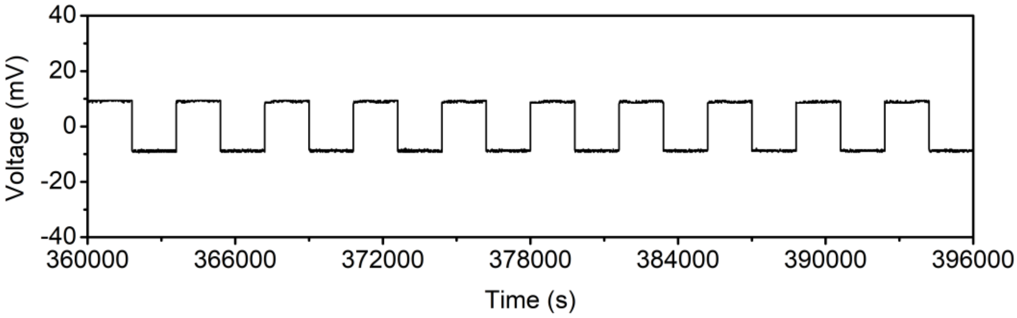

Supplementary Figure 18 | The enlarged voltage profiles of Li/$Li_{22}Sn_5$|Li/$Li_{22}Sn_5$ symmetric cells with a Li/Sn atomic ratio of 88/5 in the electrode from 100 to 110 cycles under 10 mA cm^{-2} with fixed areal capacity of 5 mAh cm^{-2}.

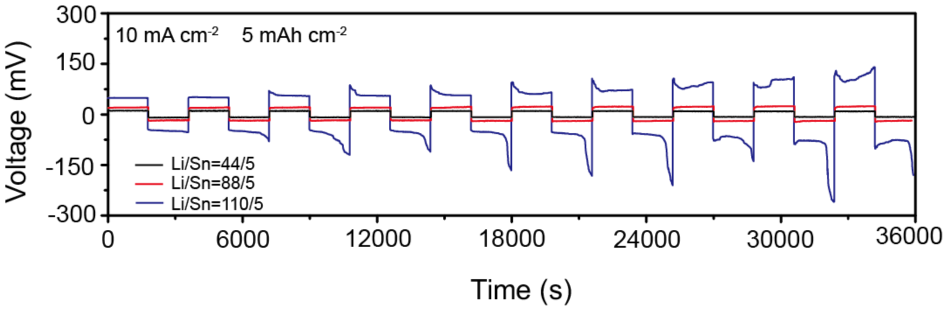

Supplementary Figure 19 | Voltage profiles of the Li/$Li_{22}Sn_5$| Li/$Li_{22}Sn_5$ cells with different Li/Sn molar ratios (44/5, 88/5, 110/5) from 1 to 10 cycles at 10 mA cm^{-2} with fixed areal capacity of 5 mAh cm^{-2}.

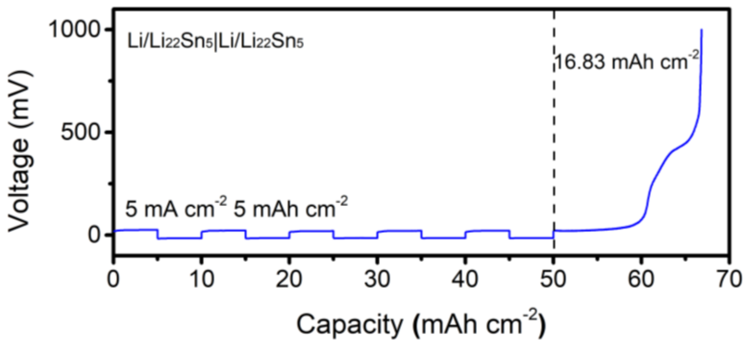

Supplementary Figure 20 | Plots of voltage *vs*. capacity for a Li/$Li_{22}Sn_5$ electrode with low metallic Li loading (~10 mAh cm^{-2}) for 5 stripping/plating cycles at 5 mA cm^{-2} with areal capacity fixed at 5 mAh cm^{-2} followed by the full Li extraction at 5 mA cm^{-2} with the cut-off voltage of 1 V (*vs.* Li^+/Li).

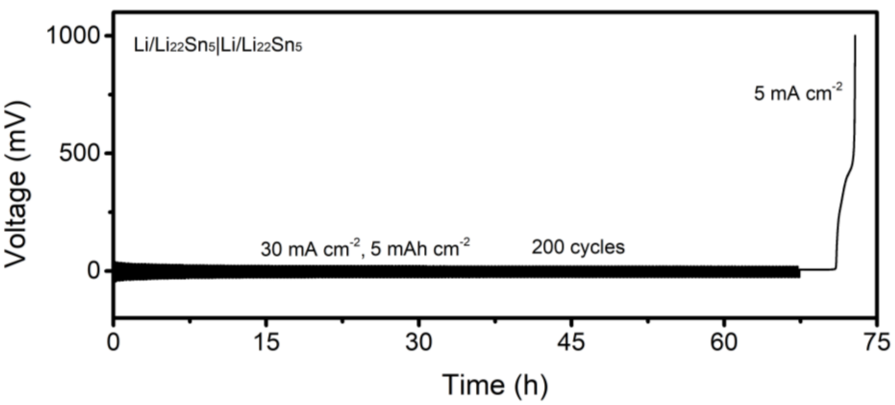

Supplementary Figure 21 | Plots of voltage *vs.* capacity for a Li/$Li_{22}Sn_5$ electrode after 200 stripping/plating cycles at 30 mA cm^{-2} with areal capacity fixed at 5 mAh cm^{-2} followed by the full Li extraction at 5 mA cm^{-2} with the cut-off voltage of 1 V (*vs.* Li^+/Li).

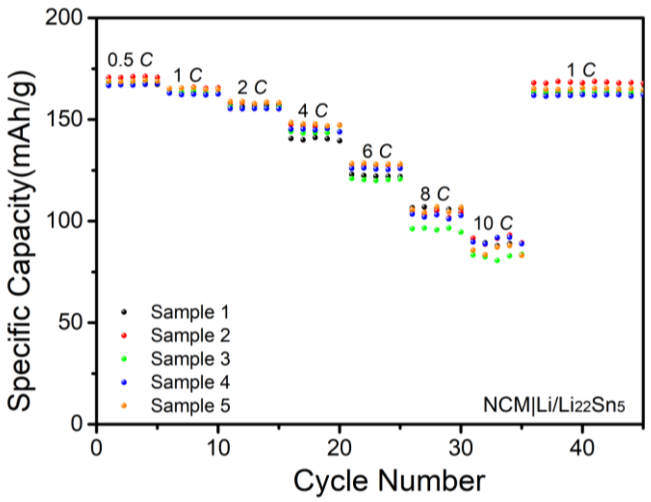

Supplementary Figure 22 | Rate capability of 5 $LiNi_{0.6}Co_{0.2}Mn_{0.2}O_2$ (NCM)|Li/$Li_{22}Sn_5$ cells with NCM loading of ~6.5 mg cm^{-2} at various rates from 0.5 to 10 *C*.

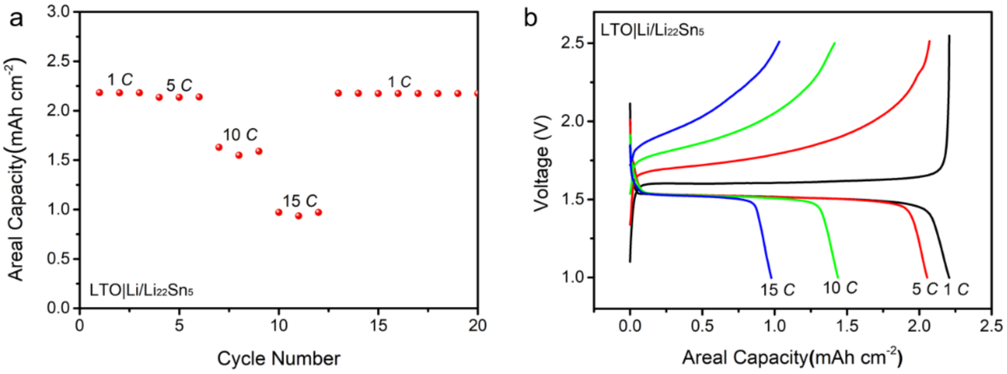

Supplementary Figure 23 | Rate capability of the $Li_4Ti_5O_{12}$ (LTO)|Li/$Li_{22}Sn_5$ cells with LTO loading of 12.5 mg cm^{-2}. (a) Areal capacity *vs.* cycle number plot. (b) Areal capacity *vs.* voltage plot. The cells were discharged at a 1 *C* and charged at various rates from 1 *C* (2.2 mA cm^{-2}) to 15 *C* (33.1 mA cm^{-2}). LTO is considered as a fast-charging electrode material. A LTO|Li/$Li_{22}Sn_5$ cell delivered a stable areal capacity of ~1 mAh cm^{-2} at 33.1 mA cm^{-2} (15 *C*), suggesting the good stability of the Li/$Li_{22}Sn_5$ electrode at ultrahigh current density.

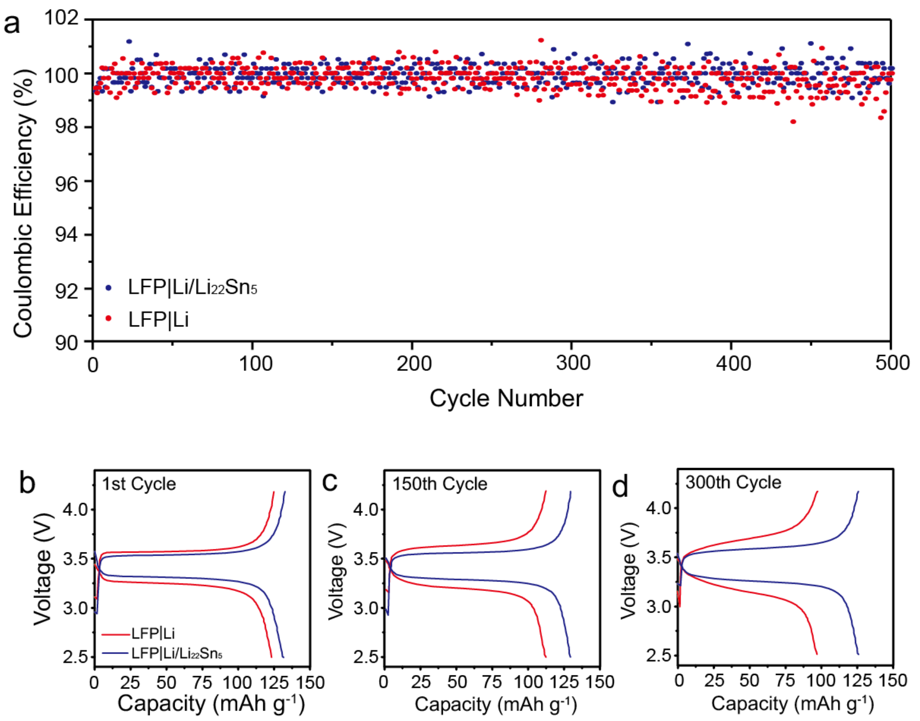

Supplementary Figure 24 | Electrochemical performance of the $LiFePO_4$ (LFP)|Li/$Li_{22}Sn_5$ and $LiFePO_4$ (LFP)|Li cells cycled at 5 *C*. (a) Coulombic efficiency and (b-d) voltage *vs.* capacity profile comparison of the LFP|Li/$Li_{22}Sn_5$ and LFP|Li cells cycled at 5 *C* for different cycles.

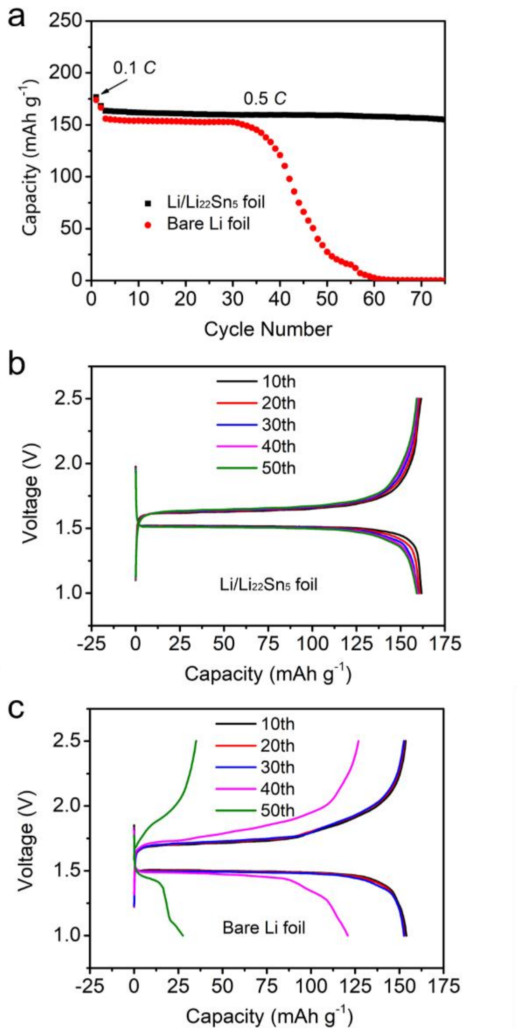

Supplementary Figure 25 | Electrochemical performance of LTO|$Li_{22}Sn_5$/Li and LTO|Li cells with a practical areal capacity of 2.8 mAh cm^{-2} cycled at 1.4 mA cm^{-2}. (**a**) Capacity *vs.* cycle number profile of LTO|$Li_{22}Sn_5$/Li and LTO|Li cells. (**b**,**c**) Voltage *vs.* capacity profiles of LTO|$Li_{22}Sn_5$/Li (b) and LTO|Li cell (**c**) over cycling. LTO electrode comprised of 90% active materials, 5% polyvinylidene fluoride (PVDF) and 5% carbon black.

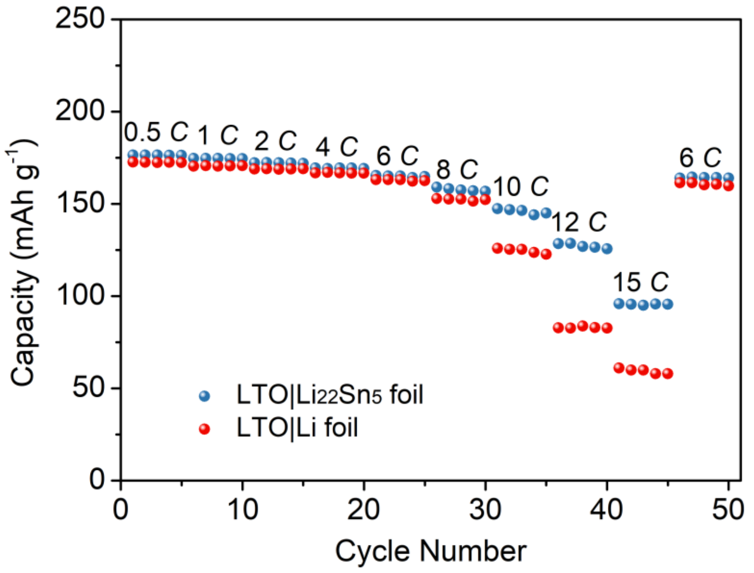

Supplementary Figure 26 | Rate capability of the LTO|Li/$Li_{22}Sn_5$ and LTO|Li foil cells with LTO loading of ~6 mg cm^{-2} at various rates from 0.5 to 15 *C*. The Li|LTO full cell delivered capacities of 172 mAh g^{-1} at 0.5 *C*, 170 mAh g^{-1} at 1 *C*, 168 mAh g^{-1} at 2 *C*, 162 mAh g^{-1} at 6 *C* and 82 mAh g^{-1} at 12 *C*, while Li/$Li_{22}Sn_5$|LTO cells offered capacities of 175 mAh g^{-1} at 0.5 *C*, 174 mAh g^{-1} at 1 *C*, 172 mAh g^{-1} at 2 *C*, 165 mAh g^{-1} at 6 *C* and 128 mAh g^{-1} at 12 *C*.

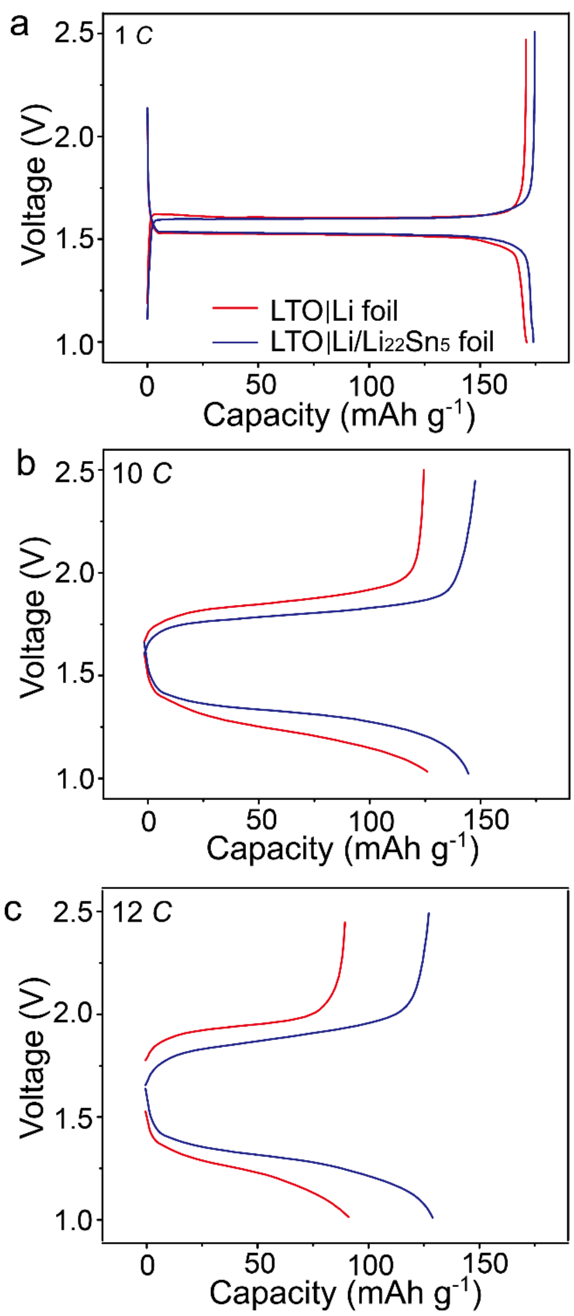

Supplementary Figure 27 | Voltage *vs.* capacity profile comparison of the LTO|Li/$Li_{22}Sn_5$ and LTO|Li foil cells at rates of 1 *C* (a), 10 *C* (b) and 12 *C* (c). Similarly to the cells using Li/$Li_{22}Sn_5$ anodes paired with $LiNi_{0.6}Co_{0.2}Mn_{0.2}O_2$ (NCM) cathodes, the rate performance of the LTO|Li/$Li_{22}Sn_5$ cells outperformed the LTO|Li foil cells.

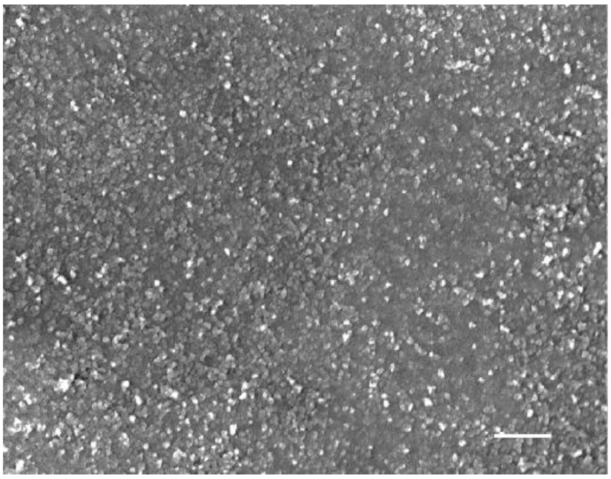

Supplementary Figure 28 | Top-view SEM images of the Li/$Li_{22}Sn_5$ foil after stripping 5 mAh cm^{-2} of lithium under 5 mA cm^{-2}(scale bar, 1 μm).

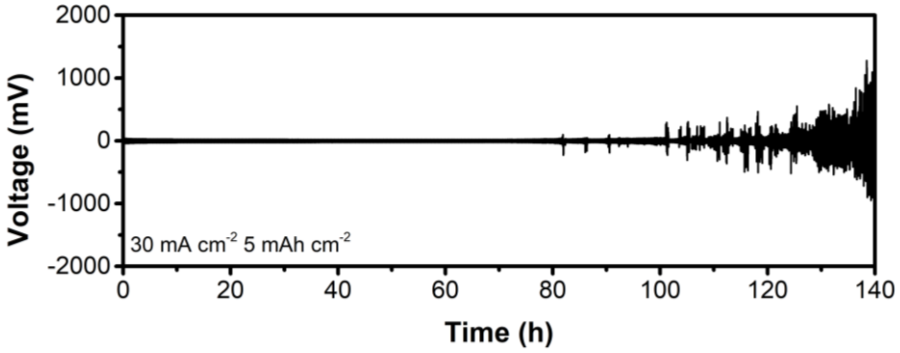

Supplementary Figure 29 | Galvanostatic lithium plating/stripping cycling and voltage profiles of the Li/$Li_{22}Sn_5$|Li/$Li_{22}Sn_5$ cell at 30 mA cm^{-2} with fixed areal capacity of 5 mAh cm^{-2}.

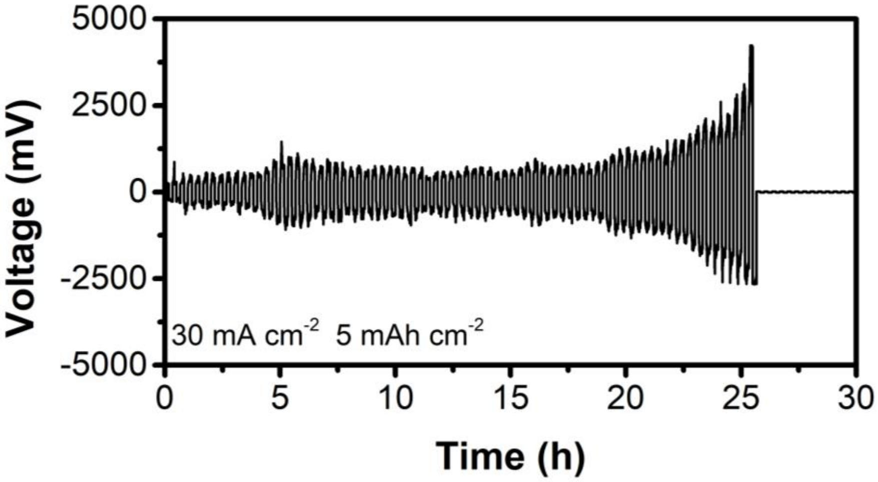

Supplementary Figure 30 | Galvanostatic lithium plating/stripping cycling of the Li|Li symmetric cell at 30 mA cm^{-2} and 5 mAh cm^{-2}.

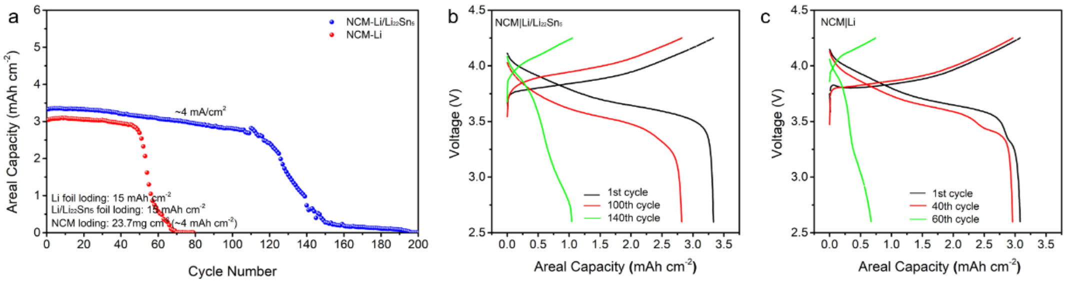

Supplementary Figure 31 | Electrochemical performance of $LiNi_{0.6}Co_{0.2}Mn_{0.2}O_2$ (NCM)|Li/$Li_{22}Sn_5$ cells. (a) Capacity *vs.* cycle number profiles of the NCM|Li/$Li_{22}Sn_5$ and NCM|Li cells with high NCM mass loading (~23.7 mg cm^{-2}) and low areal capacity ratio of negative to positive electrodes (N/P ratio, 3.75, 15 mAh cm^{-2} for the anode). (b, c) Voltage *vs.* capacity profiles of the NCM|Li/$Li_{22}Sn_5$ (b) and NCM|Li (c) cells for various cycles cycled at 4 mA cm^{-2}.

Supplementary Note 1 | Li/$Li_{22}Sn_5$|Li/$Li_{22}Sn_5$ symmetric cells with much lower loadings (~10 mAh cm^{-2} of metallic Li) were built and electrochemical full extraction of Li from a Li/$Li_{22}Sn_5$ foil electrode was performed at 5 mA cm^{-2} with the cut-off voltage of 1 V (*vs.* Li^+/Li) after 5 stripping/plating cycles at 5 mA cm^{-2} with areal capacity fixed at 5 mAh cm^{-2} (Supplementary Fig. 20). During the process of full Li extraction, a low and flat voltage plateau close to 0 V (*vs.* Li^+/Li) was first observed, corresponding to the stripping of metallic Li. The voltage started to increase after the areal capacity reached ~10 mAh cm^{-2}, which indicated that the metallic Li in the composites was exhausted and the Li in the $Li_{22}Sn_5$ started to be extracted. With further increase of the extracted lithium-ion capacity, the voltage-capacity plots showed voltage plateaus between 0.1-0.65 V, corresponding to the extraction process of Li from $Li_{22}Sn_5$. The voltage gradually reached 1 V after the exhaustion of most of the stored lithium and an overall areal capacity of ~16.8 mAh cm^{-2} was achieved. The gradual increase in voltage after the full stripping of metallic Li for the cycled electrode verified that shorts did not take place for the Li/$Li_{22}Sn_5$|Li/$Li_{22}Sn_5$ cells during cycling.

Supplementary Note 2 | The full stripping measurement was performed at 5 mA cm^{-2} with the cut-off voltage of 1 V (*vs.* Li^+/Li) for a Li/$Li_{22}Sn_5$|Li/$Li_{22}Sn_5$ cell after 200 stripping/plating cycles at 30 mA cm^{-2} and 5 mAh cm^{-2} (Supplementary Fig. 21). The voltage rapidly reached 1 V after the exhaustion of all the stored lithium during the lithium extraction process. The sharp increase in voltage after the full stripping of metallic Li for the cycled electrode verified that short did not take place for the Li/$Li_{22}Sn_5$|Li/$Li_{22}Sn_5$ cells after 200 cycles cycled at 30 mA cm^{-2} and 5 mAh cm^{-2}, which meets the demand for high-power-density applications, such as drone.

Supplementary Note 3 | The Li/$Li_{22}Sn_5$|Li/$Li_{22}Sn_5$ cells cycled stably with low overpotential for 246 cycles (82 hours) at 30 mA cm^{-2} and 5 mAh cm^{-2} (Supplementary Figure 29) beyond the time cutoff in Fig. 2c (200 cycles, 67 hours). After 82 hours, the overpotential of the cells fluctuated widely and increased quickly. As a manually prepared foil, the microstructure of Li/$Li_{22}Sn_5$ electrode may not be absolutely homogeneous, then lithium stripping/plating and so caused electrode volume change may be nonuniform under such high current density, leading to consumption of electrolyte due to the change of electrode/electrolyte interface and even the growth of lithium dendrites in some location areas. The accumulation of above nonuniform electrode reactions eventually caused the failure of cells.

Supplementary Note 4 | The capacity of the NCM|Li/$Li_{22}Sn_5$ cell degraded quickly after 110 cycles, and the cell showed much longer cycle life but similar failure behavior in comparison to the counterpart using bare Li metal anode (Supplementary Figure 30). The slow capacity decay of the full cells in the initial 110 cycles mainly comes from the degradation of cathodes while the quick capacity decay after 110 cycles is considered to be caused by the accumulated consumption of active lithium and electrolyte. The further improvement can be achieved by using advanced electrolyte systems instead of the used regular carbonate electrolyte here.

Supplementary Note 5 | The capacity of the NCM|Li/$Li_{22}Sn_5$ cell degraded quickly after 110 cycles, and the cell showed much longer cycle life but similar failure behavior in comparison to the counterpart using bare Li metal anode (Supplementary Fig. 31). The slow capacity decay of the full cells in the initial 110 cycles mainly comes from the degradation of cathodes while the quick capacity decay after 110 cycles is considered to be caused by the accumulated consumption of active lithium and electrolyte.
