# Supplementary material for: Mechanical rolling formation of interpenetrated lithium metal/lithium tin alloy foil for ultrahigh-rate battery anode
Source: Nat Commun. 2020 Feb 11;11:829. doi: 10.1038/s41467-020-14550-3 (PMC7012843; doi:10.1038/s41467-020-14550-3)
Supplement: Supplementary file 2 — Description of Additional Supplementary Files [file 41467_2020_14550_MOESM2_ESM.pdf]

Description of Additional Supplementary Files

Supplementary Movie 1 | *In situ* optical microscopy investigation of the electrolyte and electrode interfaces for the Li|Li symmetric cells during lithium plating process (1 mA cm^{-2} at 10 mAh cm^{-2}).

Supplementary Movie 2 | *In situ* optical microscopy investigation of the electrolyte and electrode interfaces for the Li/$Li_{22}Sn_5$|Li/$Li_{22}Sn_5$ symmetric cells during lithium plating process (1 mA cm^{-2} at 10 mAh cm^{-2}).
